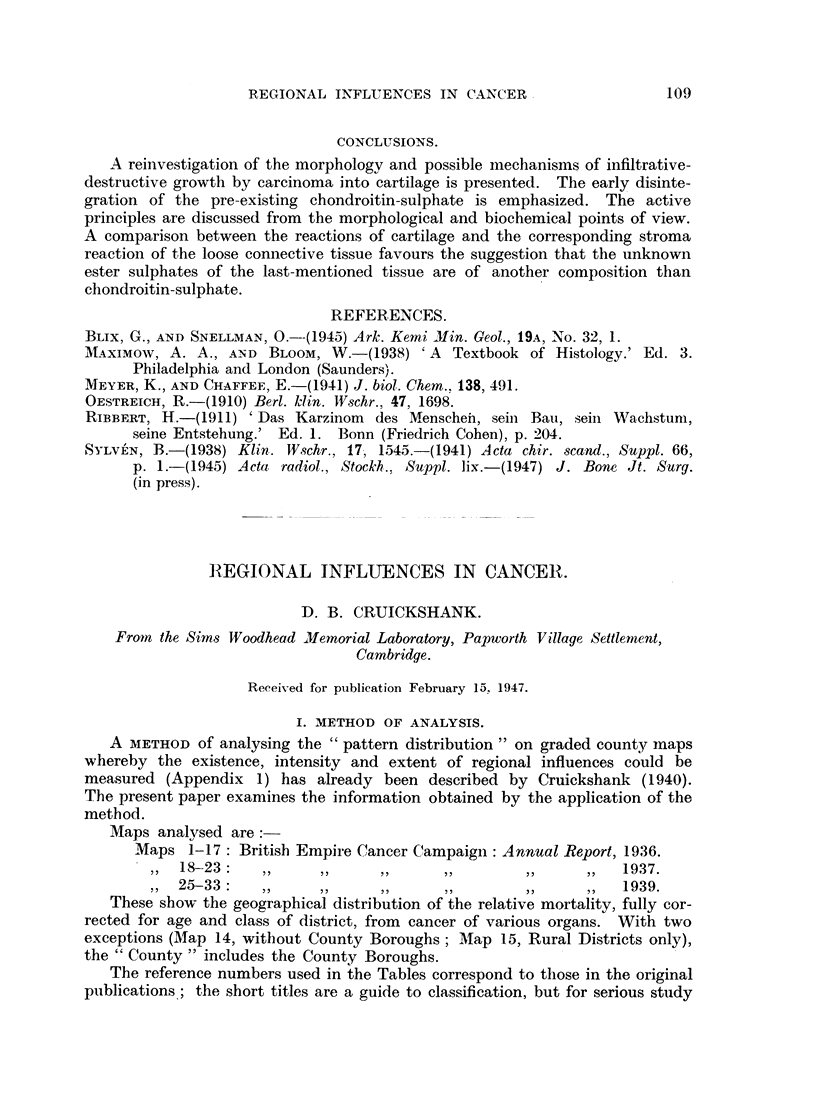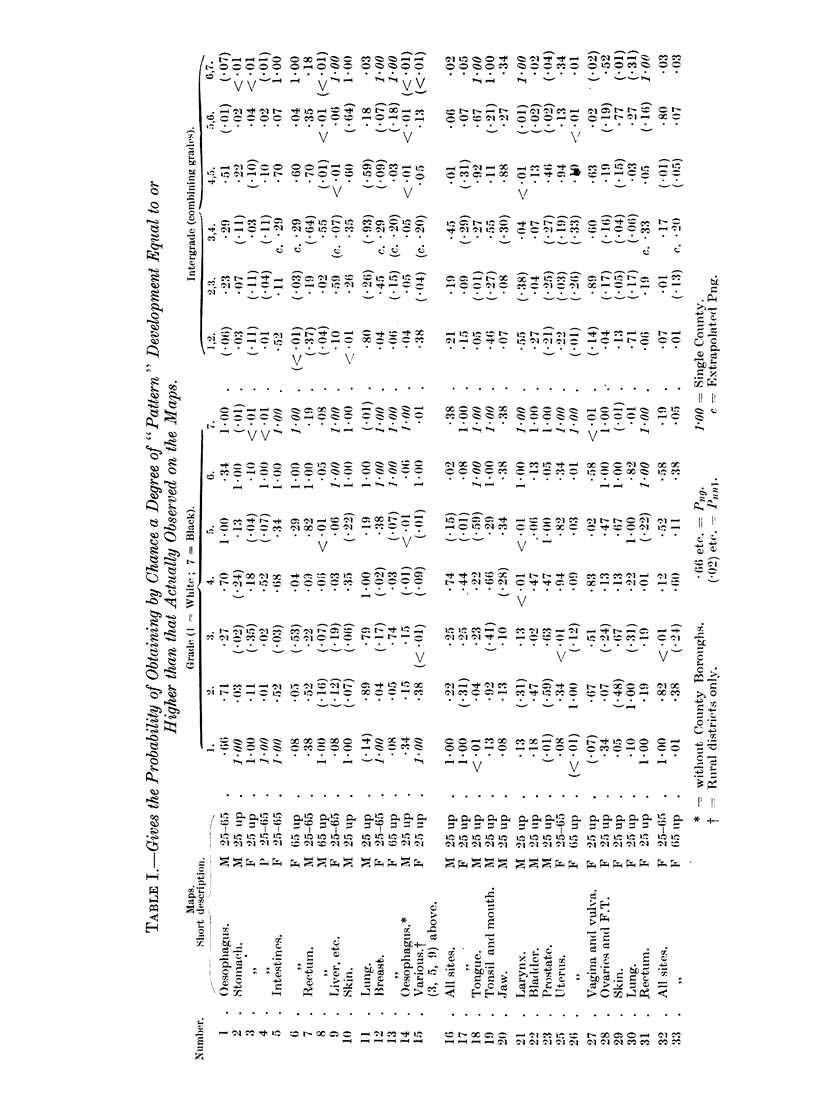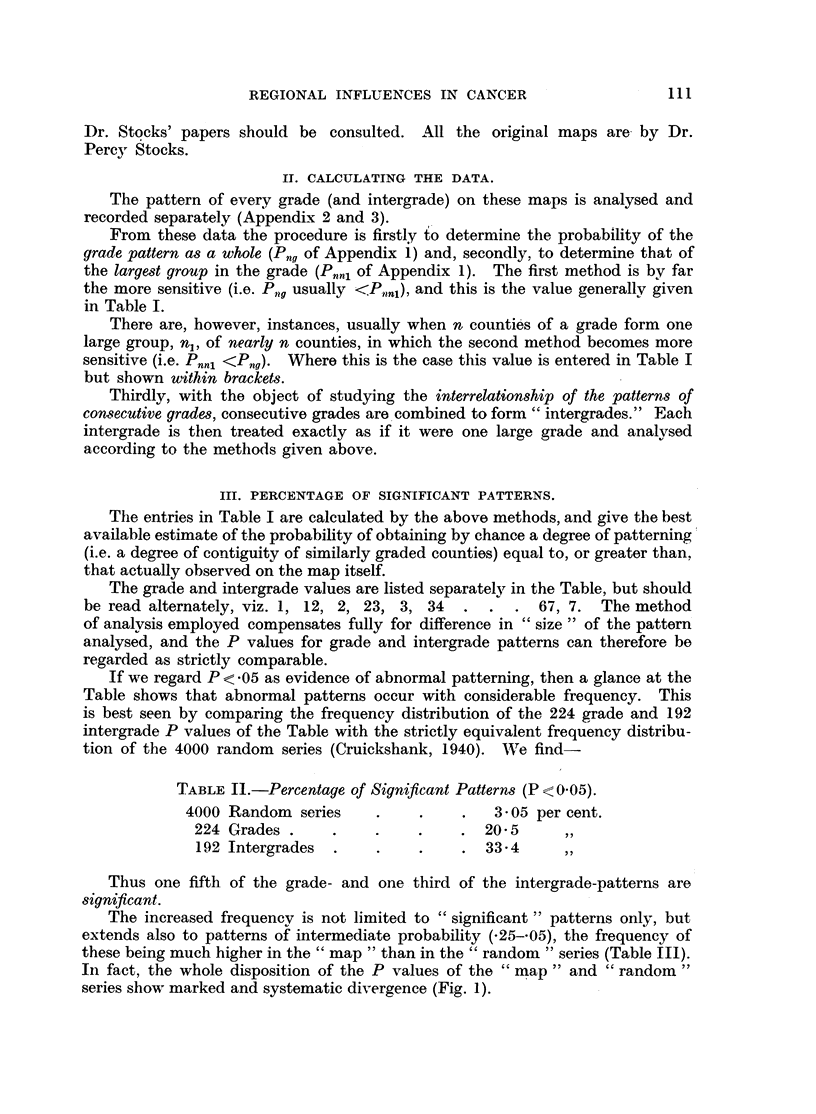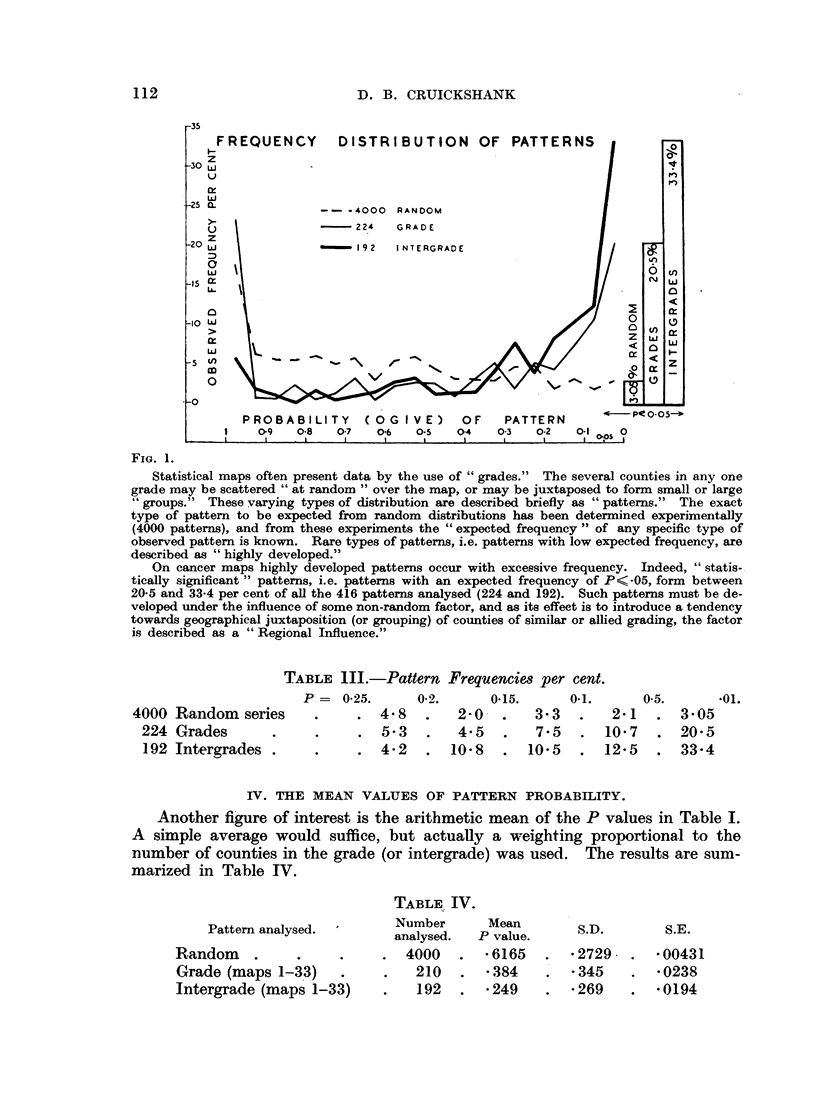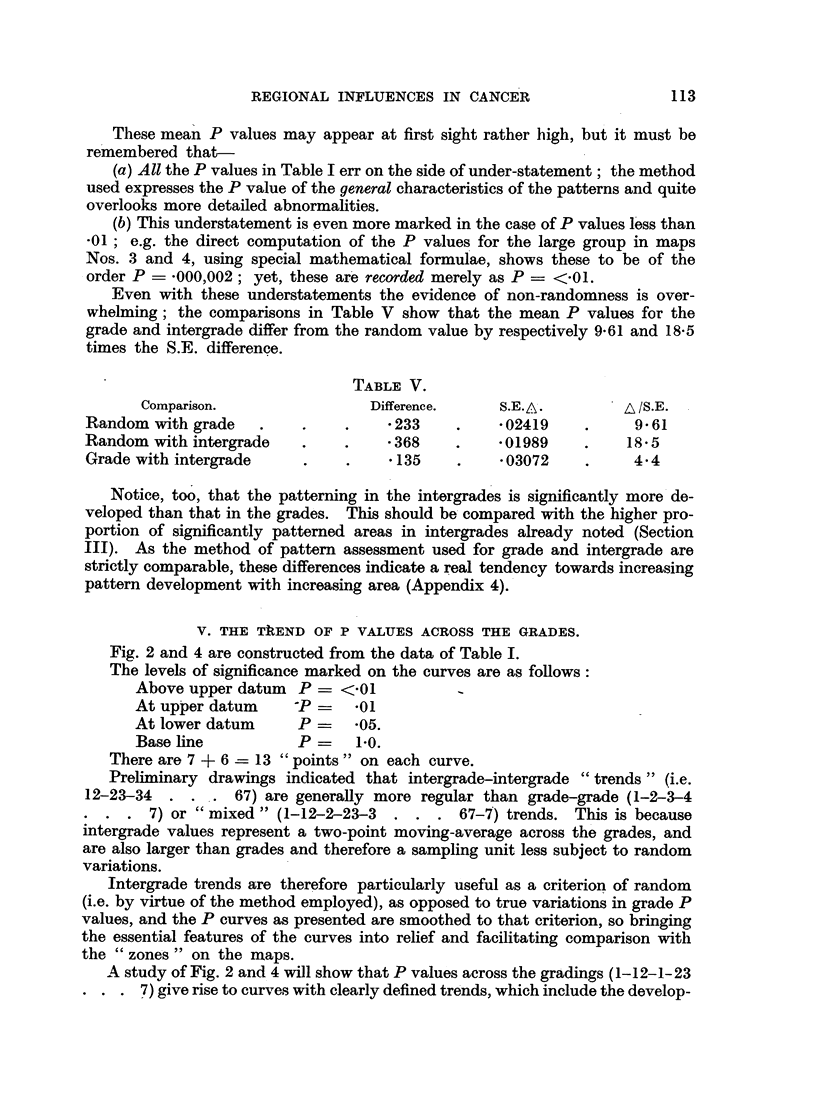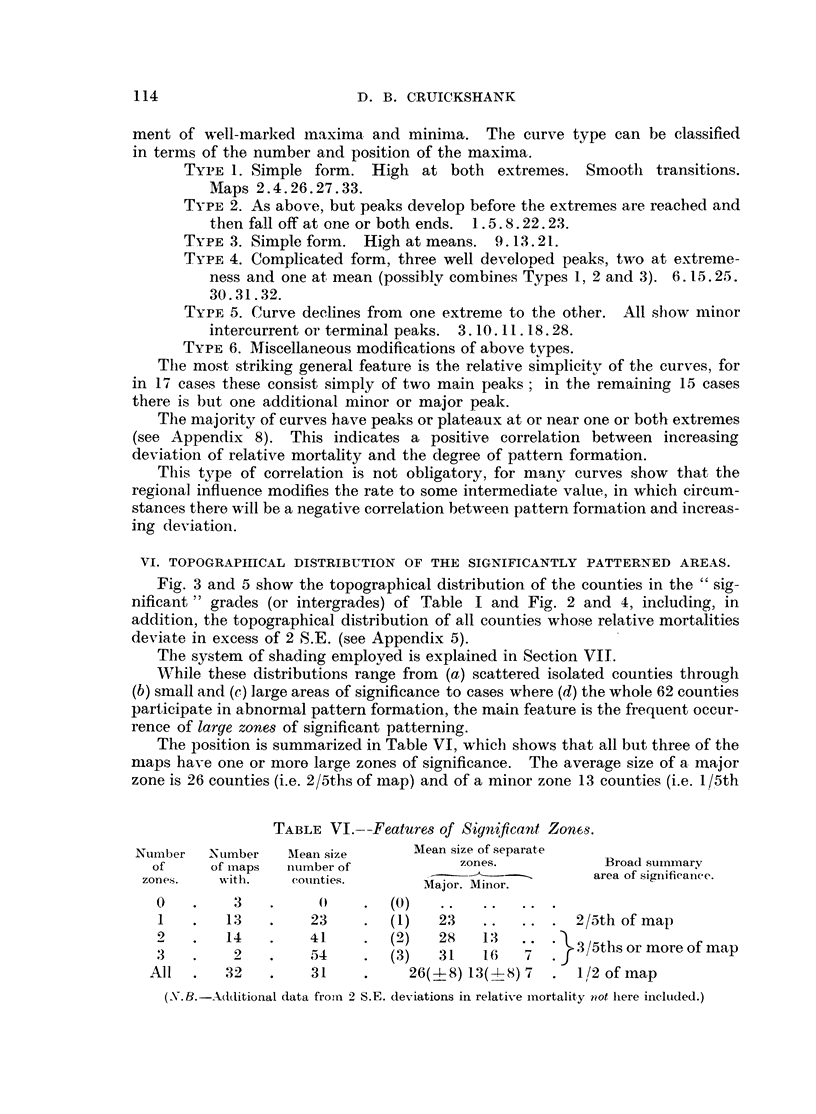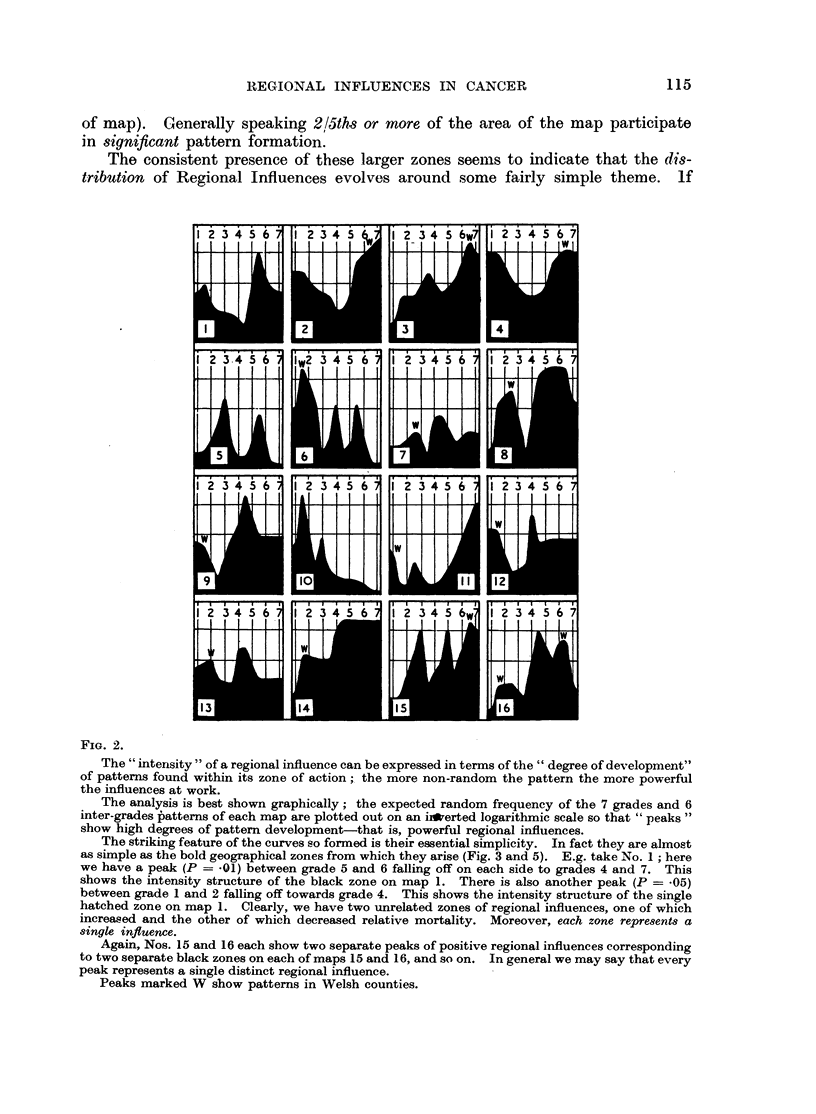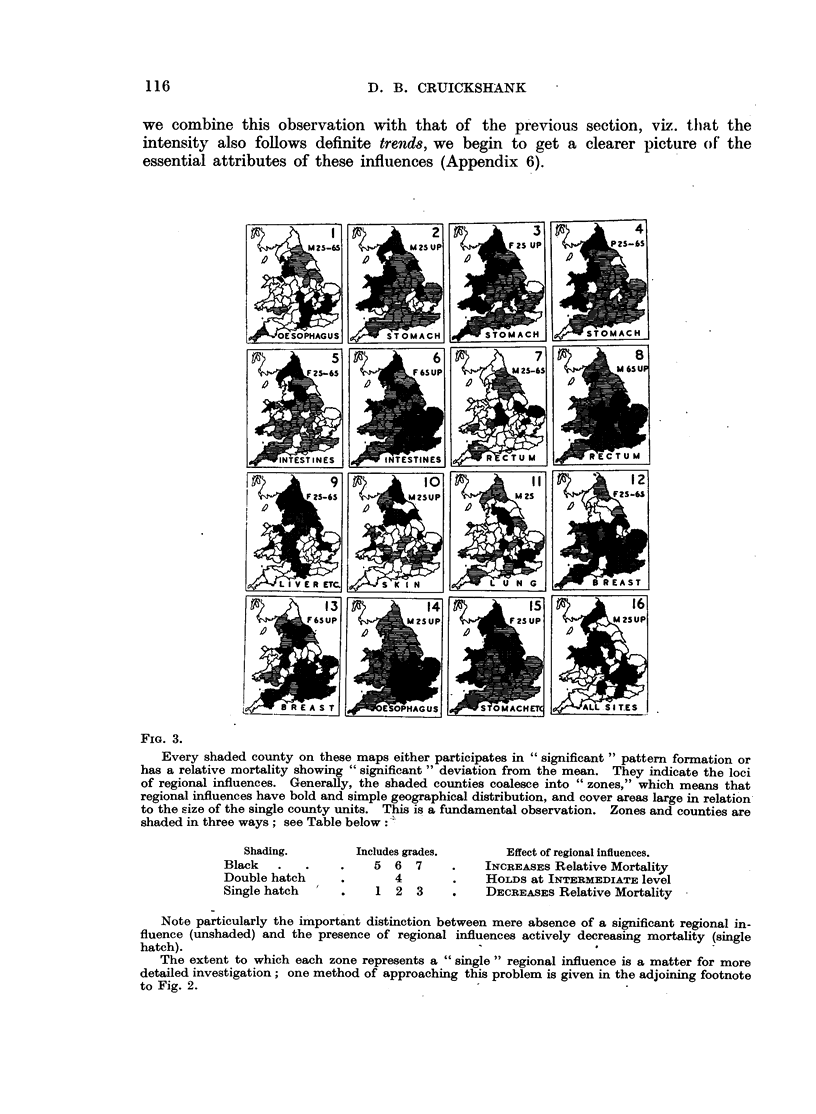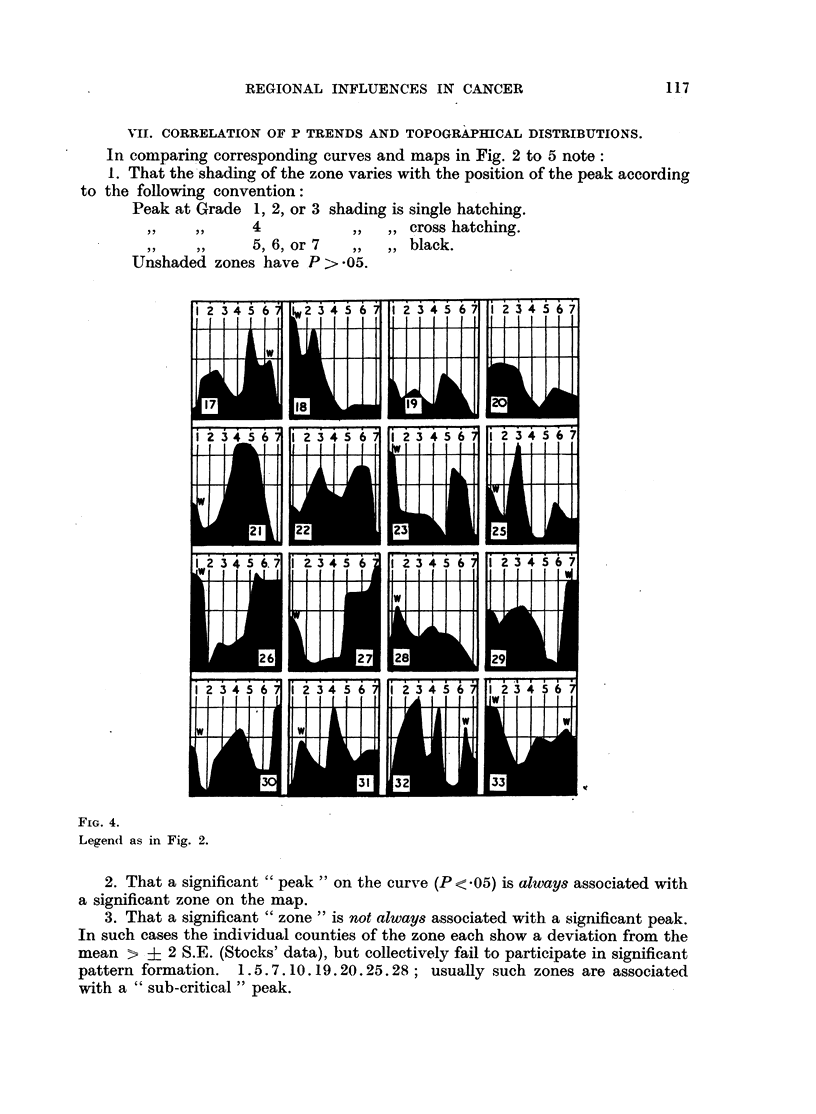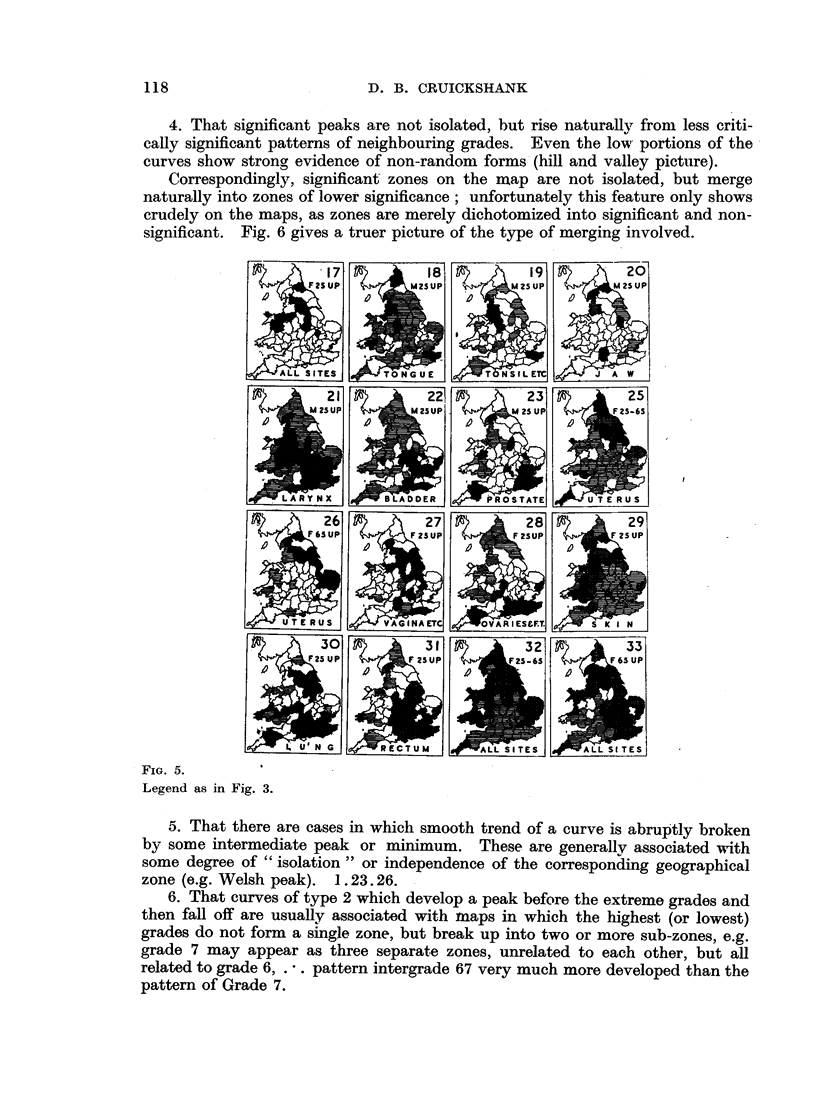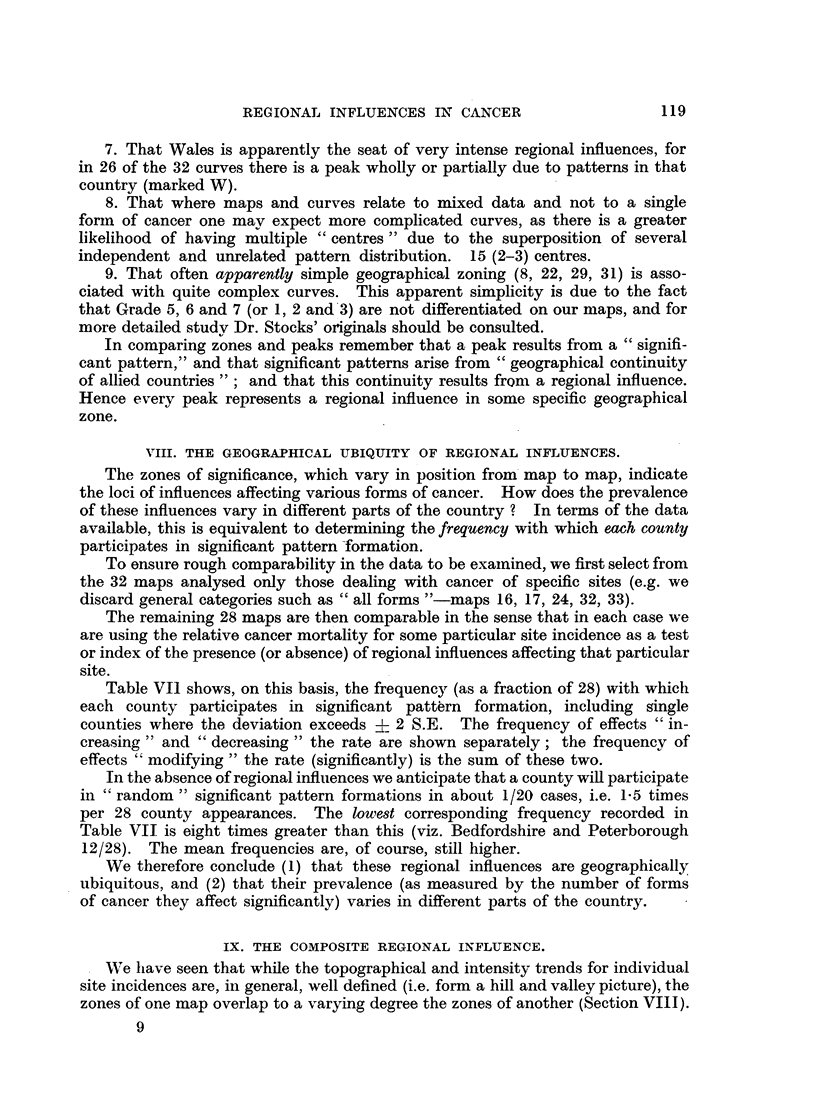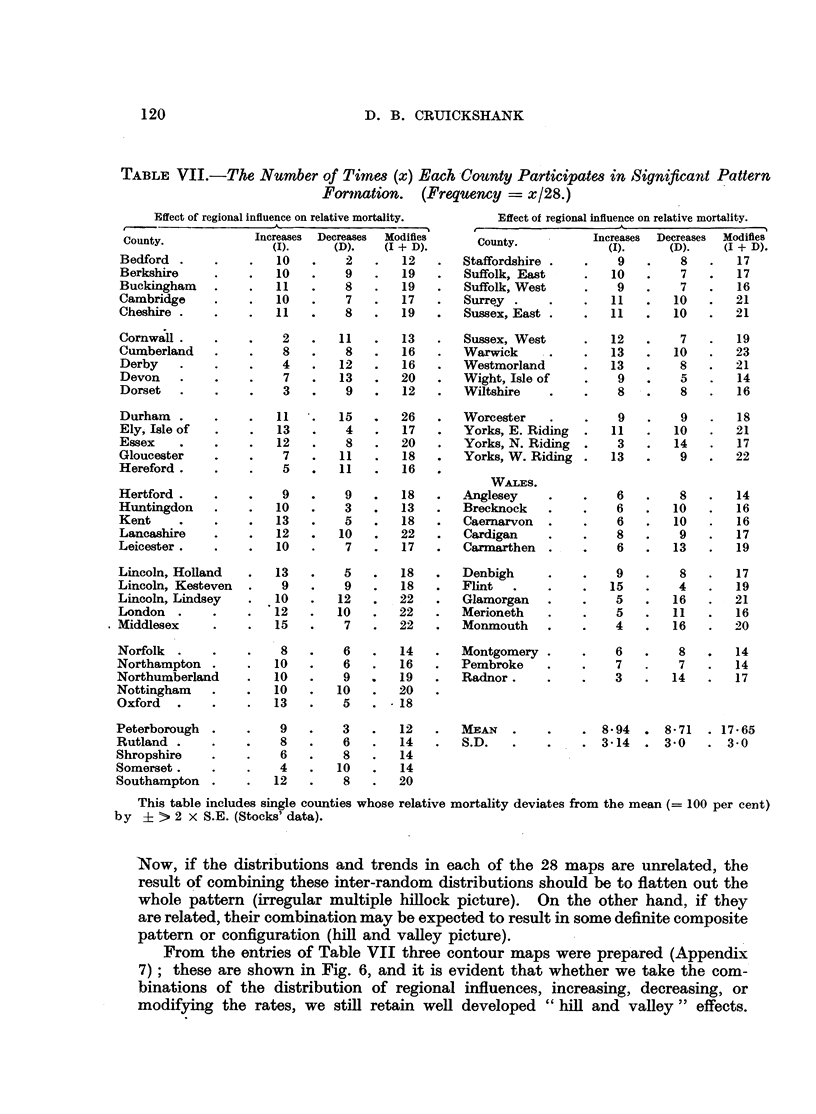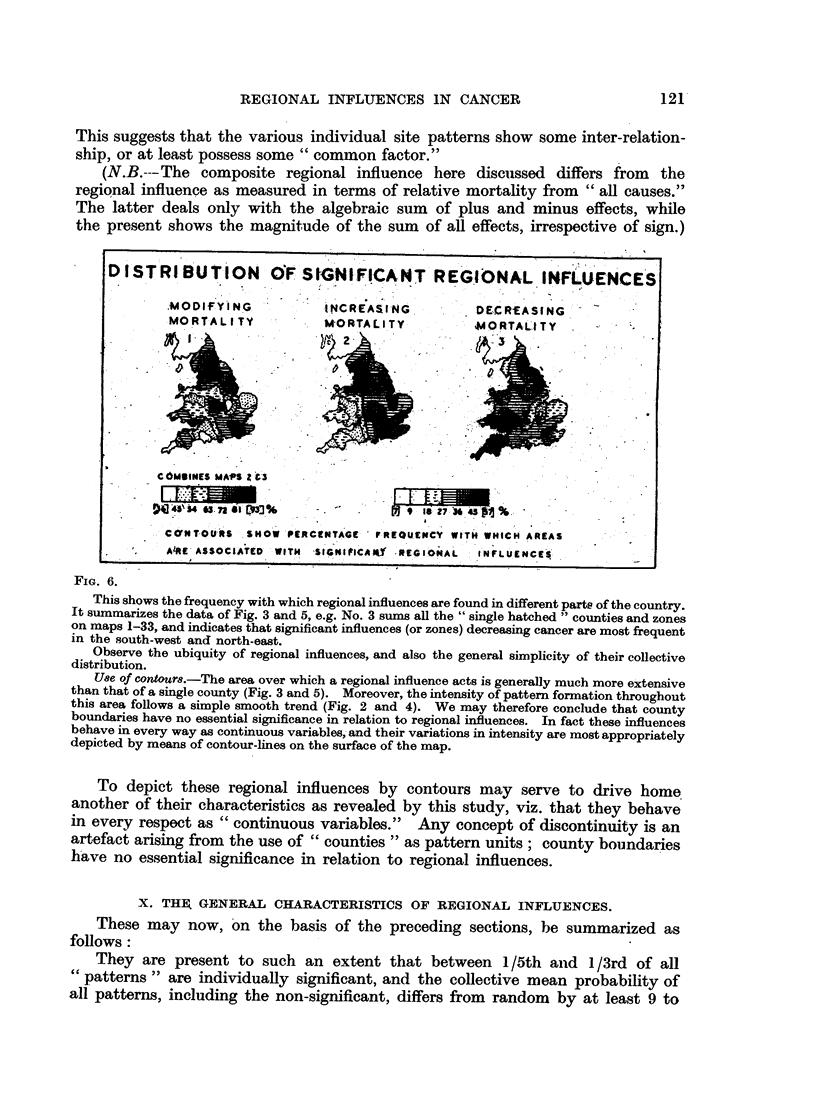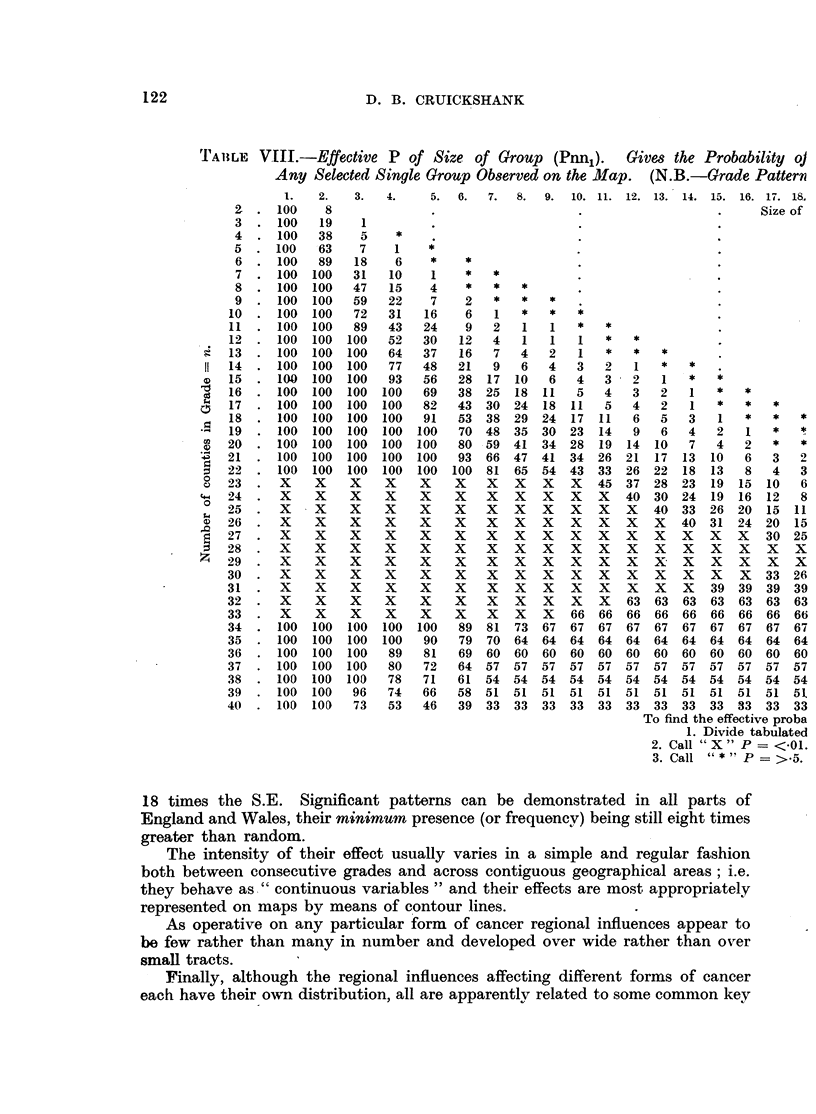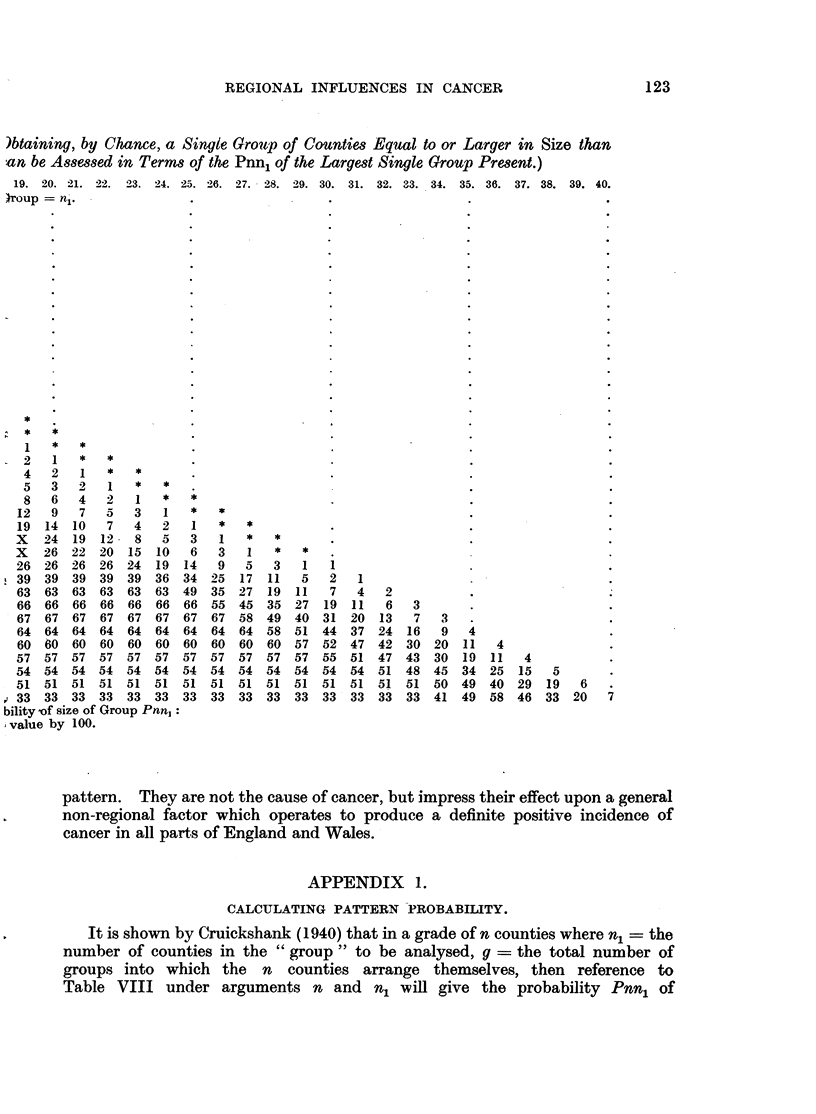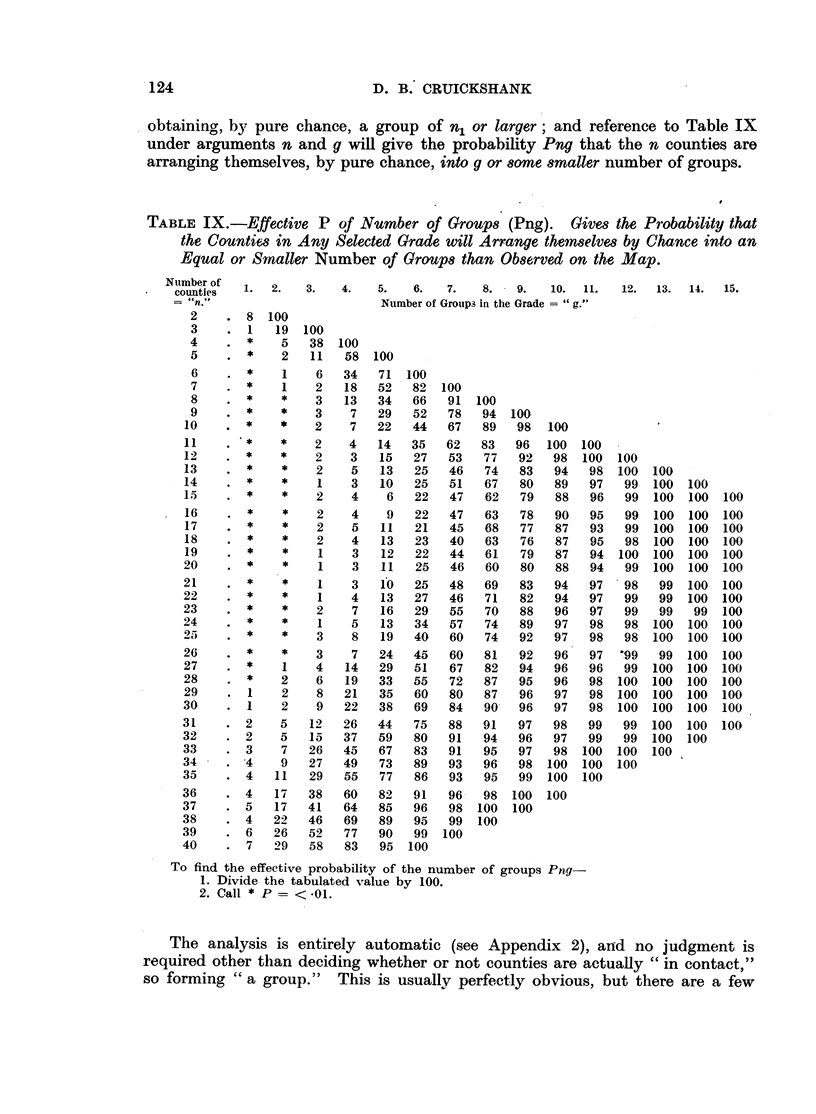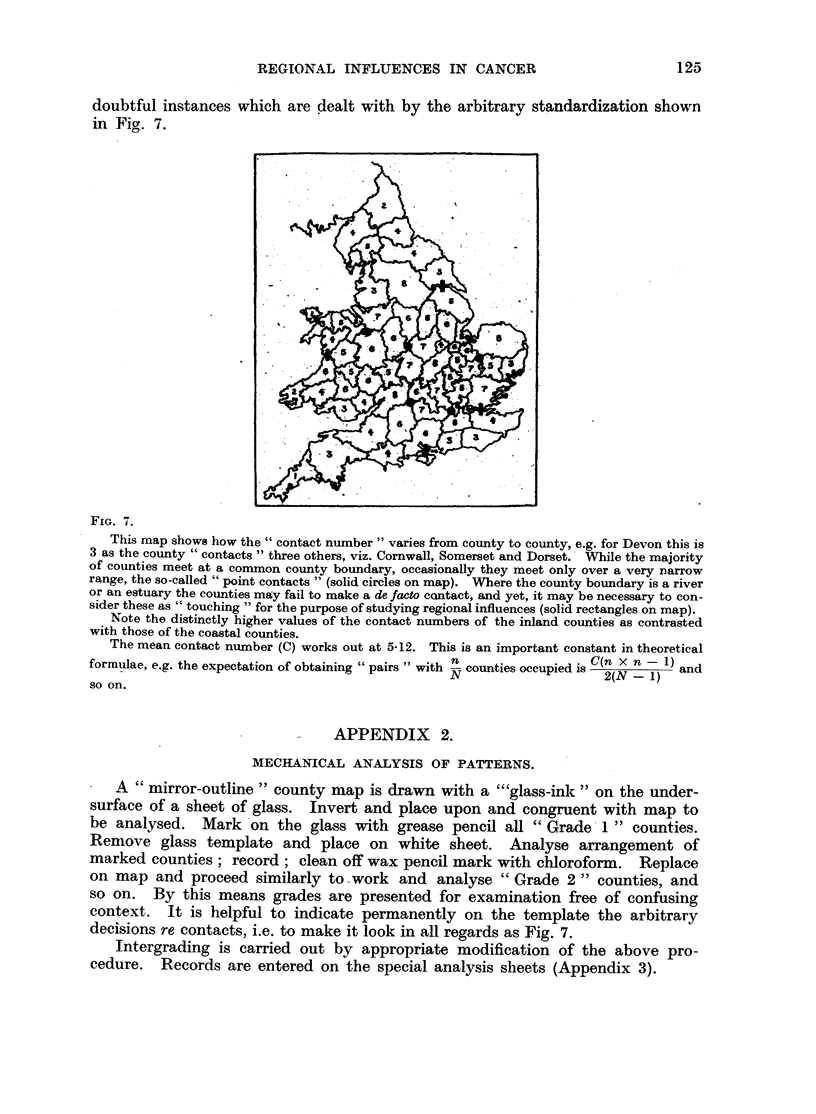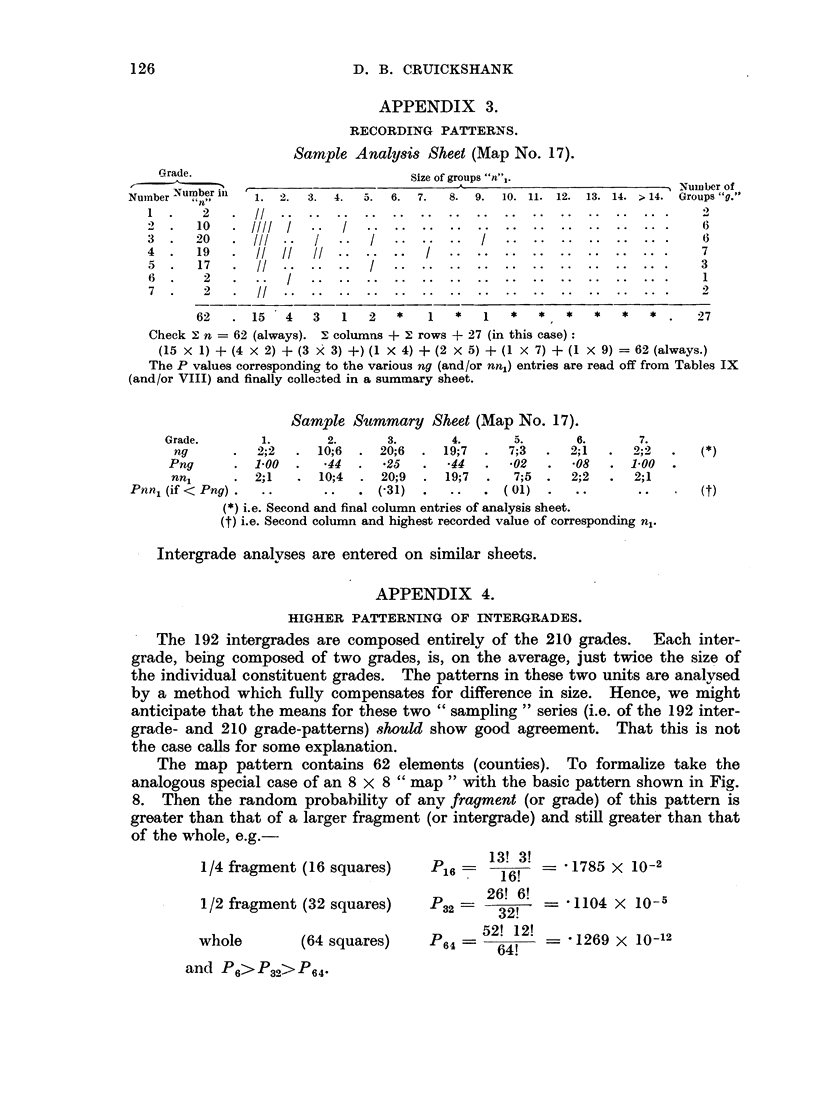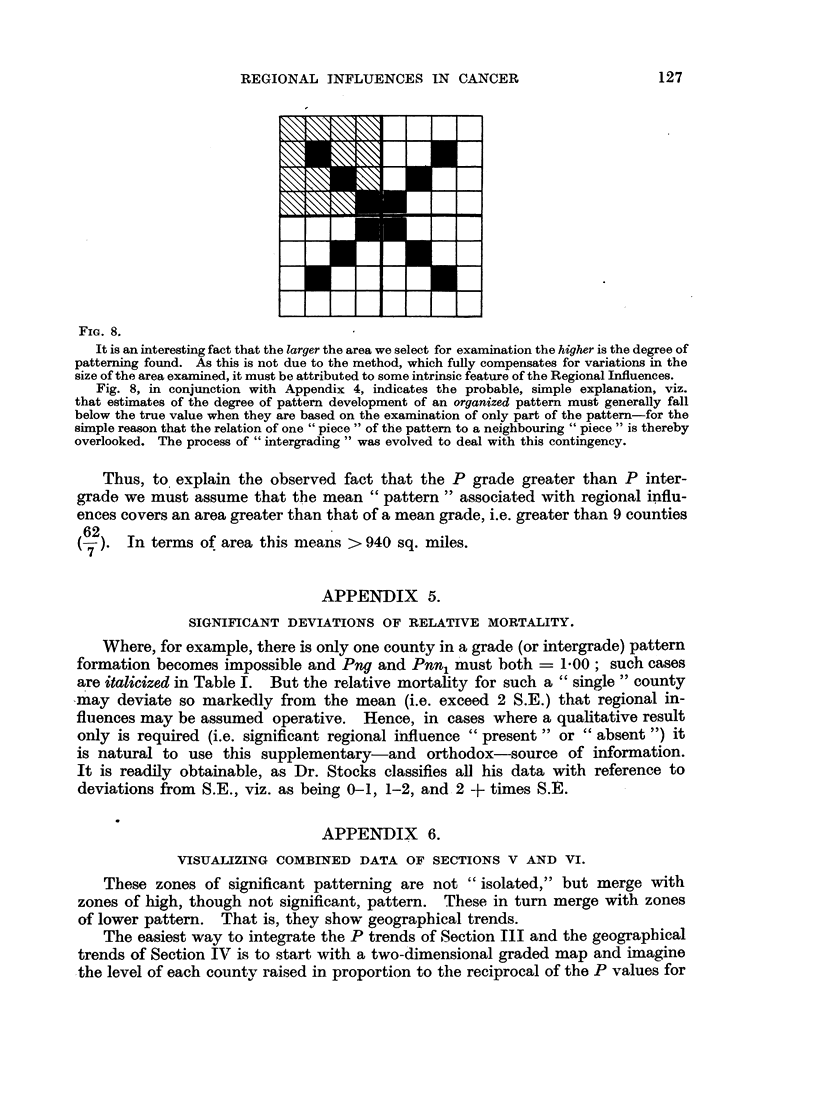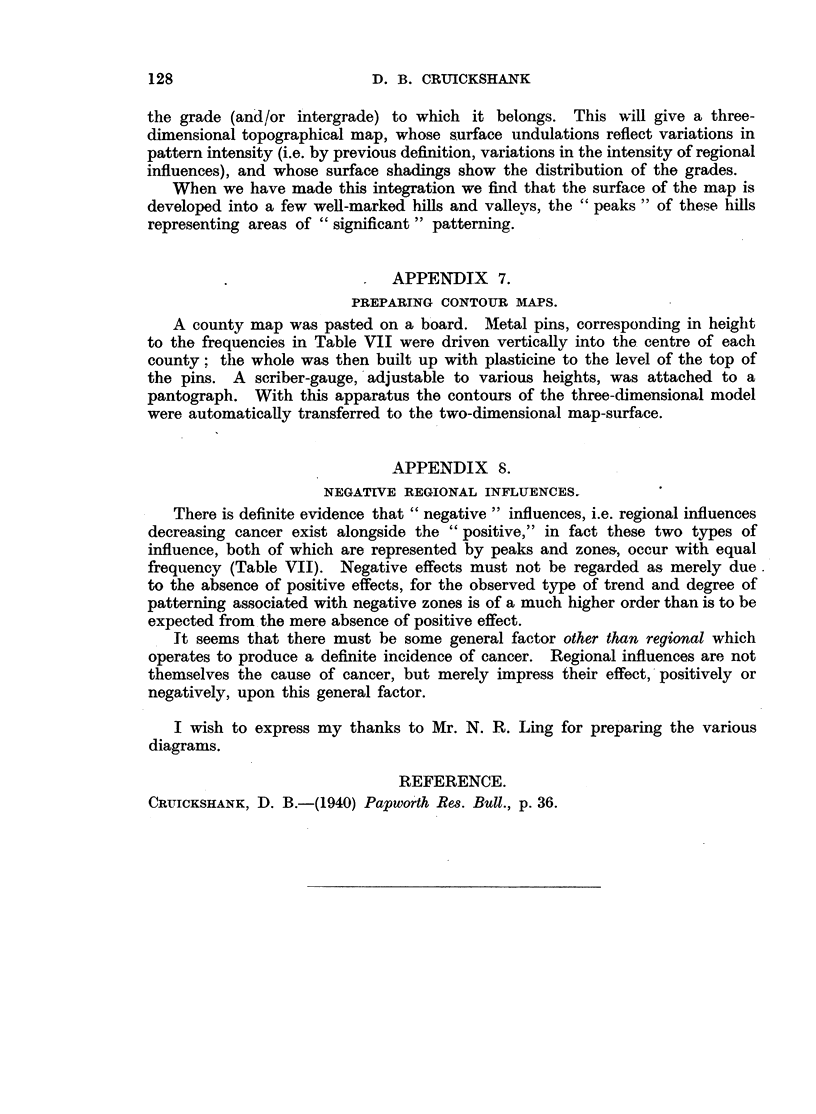# Regional Influences in Cancer

**DOI:** 10.1038/bjc.1947.14

**Published:** 1947-06

**Authors:** D. B. Cruickshank


REFERENCES.

### REGIONAL INFLUENCES IN CANCER.

D. B. CRUICKSHANK.

*From the Sims Woodhead Memorial Laboratory, Papworth Village Settlement, Cambridge.*

Received for publication February 15, 1947.

### I. METHOD OF ANALYSIS.

A METHOD of analysing the "pattern distribution" on graded county maps whereby the existence, intensity and extent of regional influences could be measured (Appendix 1) has already been described by Cruickshank (1940). The present paper examines the information obtained by the application of the method.

Maps analysed are :—

Maps 1–17 : British Empire Cancer Campaign : *Annual Report*, 1936.
,, 18–23 : ,, ,, ,, ,, ,, ,, 1937.
,, 25–33 : ,, ,, ,, ,, ,, ,, 1939.

These show the geographical distribution of the relative mortality, fully corrected for age and class of district, from cancer of various organs. With two exceptions (Map 14, without County Boroughs ; Map 15, Rural Districts only), the "County" includes the County Boroughs.

The reference numbers used in the Tables correspond to those in the original publications ; the short titles are a guide to classification, but for serious study

Dr. Stocks' papers should be consulted. All the original maps are by Dr. Percy Stocks.

### II. CALCULATING THE DATA.

The pattern of every grade (and intergrade) on these maps is analysed and recorded separately (Appendix 2 and 3).

From these data the procedure is firstly to determine the probability of the *grade pattern as a whole* ($P_{ng}$ of Appendix 1) and, secondly, to determine that of the *largest group* in the grade ($P_{nn1}$ of Appendix 1). The first method is by far the more sensitive (i.e. $P_{ng}$ usually $< P_{nn1}$), and this is the value generally given in Table I.

There are, however, instances, usually when $n$ counties of a grade form one large group, $n_1$, of *nearly* $n$ counties, in which the second method becomes more sensitive (i.e. $P_{nn1} < P_{ng}$). Where this is the case this value is entered in Table I but shown *within brackets.*

Thirdly, with the object of studying the *interrelationship of the patterns of consecutive grades,* consecutive grades are combined to form " intergrades." Each intergrade is then treated exactly as if it were one large grade and analysed according to the methods given above.

### III. PERCENTAGE OF SIGNIFICANT PATTERNS.

The entries in Table I are calculated by the above methods, and give the best available estimate of the probability of obtaining by chance a degree of patterning (i.e. a degree of contiguity of similarly graded counties) equal to, or greater than, that actually observed on the map itself.

The grade and intergrade values are listed separately in the Table, but should be read alternately, viz. 1, 12, 2, 23, 3, 34 . . . 67, 7. The method of analysis employed compensates fully for difference in " size " of the pattern analysed, and the $P$ values for grade and intergrade patterns can therefore be regarded as strictly comparable.

If we regard $P < \cdot 05$ as evidence of abnormal patterning, then a glance at the Table shows that abnormal patterns occur with considerable frequency. This is best seen by comparing the frequency distribution of the 224 grade and 192 intergrade $P$ values of the Table with the strictly equivalent frequency distribution of the 4000 random series (Cruickshank, 1940). We find—

TABLE II.—*Percentage of Significant Patterns* ($P < 0 \cdot 05$).

| | | |
|---|---|---|
| 4000 | Random series . . . | 3·05 per cent. |
| 224 | Grades . . . . . | 20·5 ,, |
| 192 | Intergrades . . . . | 33·4 ,, |

Thus one fifth of the grade- and one third of the intergrade-patterns are *significant.*

The increased frequency is not limited to " significant " patterns only, but extends also to patterns of intermediate probability (·25–·05), the frequency of these being much higher in the " map " than in the " random " series (Table III). In fact, the whole disposition of the $P$ values of the " map " and " random " series show marked and systematic divergence (Fig. 1).

Fig. 1.

Statistical maps often present data by the use of " grades." The several counties in any one grade may be scattered " at random " over the map, or may be juxtaposed to form small or large " groups." These varying types of distribution are described briefly as " patterns." The exact type of pattern to be expected from random distributions has been determined experimentally (4000 patterns), and from these experiments the " expected frequency " of any specific type of observed pattern is known. Rare types of patterns, i.e. patterns with low expected frequency, are described as " highly developed."

On cancer maps highly developed patterns occur with excessive frequency. Indeed, " statistically significant " patterns, i.e. patterns with an expected frequency of $P \leqslant \cdot 05$, form between 20·5 and 33·4 per cent of all the 416 patterns analysed (224 and 192). Such patterns must be developed under the influence of some non-random factor, and as its effect is to introduce a tendency towards geographical juxtaposition (or grouping) of counties of similar or allied grading, the factor is described as a " Regional Influence."

Table III.—*Pattern Frequencies per cent.*

| *P* = | 0·25. | 0·2. | 0·15. | 0·1. | 0·5. | ·01. |
|---|---|---|---|---|---|---|
| 4000 Random series | 4·8 | 2·0 | 3·3 | 2·1 | 3·05 | |
| 224 Grades | 5·3 | 4·5 | 7·5 | 10·7 | 20·5 | |
| 192 Intergrades | 4·2 | 10·8 | 10·5 | 12·5 | 33·4 | |

### IV. THE MEAN VALUES OF PATTERN PROBABILITY.

Another figure of interest is the arithmetic mean of the *P* values in Table I. A simple average would suffice, but actually a weighting proportional to the number of counties in the grade (or intergrade) was used. The results are summarized in Table IV.

Table IV.

| Pattern analysed. | Number analysed. | Mean *P* value. | S.D. | S.E. |
|---|---|---|---|---|
| Random | 4000 | ·6165 | ·2729 | ·00431 |
| Grade (maps 1–33) | 210 | ·384 | ·345 | ·0238 |
| Intergrade (maps 1–33) | 192 | ·249 | ·269 | ·0194 |

These mean $P$ values may appear at first sight rather high, but it must be remembered that—

(*a*) *All* the $P$ values in Table I err on the side of under-statement ; the method used expresses the $P$ value of the *general* characteristics of the patterns and quite overlooks more detailed abnormalities.

(*b*) This understatement is even more marked in the case of $P$ values less than ·01 ; e.g. the direct computation of the $P$ values for the large group in maps Nos. 3 and 4, using special mathematical formulae, shows these to be of the order $P = \cdot 000{,}002$ ; yet, these are *recorded* merely as $P = < \cdot 01$.

Even with these understatements the evidence of non-randomness is overwhelming ; the comparisons in Table V show that the mean $P$ values for the grade and intergrade differ from the random value by respectively 9·61 and 18·5 times the S.E. difference.

TABLE V.

| Comparison. | Difference. | S.E.$\triangle$. | $\triangle$/S.E. |
|---|---|---|---|
| Random with grade | ·233 | ·02419 | 9·61 |
| Random with intergrade | ·368 | ·01989 | 18·5 |
| Grade with intergrade | ·135 | ·03072 | 4·4 |

Notice, too, that the patterning in the intergrades is significantly more developed than that in the grades. This should be compared with the higher proportion of significantly patterned areas in intergrades already noted (Section III). As the method of pattern assessment used for grade and intergrade are strictly comparable, these differences indicate a real tendency towards increasing pattern development with increasing area (Appendix 4).

### V. THE TREND OF P VALUES ACROSS THE GRADES.

Fig. 2 and 4 are constructed from the data of Table I.

The levels of significance marked on the curves are as follows :

Above upper datum $P = < \cdot 01$
At upper datum $P = \cdot 01$
At lower datum $P = \cdot 05$.
Base line $P = 1 \cdot 0$.

There are $7 + 6 = 13$ " points " on each curve.

Preliminary drawings indicated that intergrade–intergrade " trends " (i.e. 12–23–34 . . . 67) are generally more regular than grade–grade (1–2–3–4 . . . 7) or " mixed " (1–12–2–23–3 . . . 67–7) trends. This is because intergrade values represent a two-point moving-average across the grades, and are also larger than grades and therefore a sampling unit less subject to random variations.

Intergrade trends are therefore particularly useful as a criterion of random (i.e. by virtue of the method employed), as opposed to true variations in grade $P$ values, and the $P$ curves as presented are smoothed to that criterion, so bringing the essential features of the curves into relief and facilitating comparison with the " zones " on the maps.

A study of Fig. 2 and 4 will show that $P$ values across the gradings (1–12–1–23 . . . 7) give rise to curves with clearly defined trends, which include the development of well-marked maxima and minima. The curve type can be classified in terms of the number and position of the maxima.

TYPE 1. Simple form. High at both extremes. Smooth transitions. Maps 2.4.26.27.33.

TYPE 2. As above, but peaks develop before the extremes are reached and then fall off at one or both ends. 1.5.8.22.23.

TYPE 3. Simple form. High at means. 9.13.21.

TYPE 4. Complicated form, three well developed peaks, two at extremeness and one at mean (possibly combines Types 1, 2 and 3). 6.15.25. 30.31.32.

TYPE 5. Curve declines from one extreme to the other. All show minor intercurrent or terminal peaks. 3.10.11.18.28.

TYPE 6. Miscellaneous modifications of above types.

The most striking general feature is the relative simplicity of the curves, for in 17 cases these consist simply of two main peaks ; in the remaining 15 cases there is but one additional minor or major peak.

The majority of curves have peaks or plateaux at or near one or both extremes (see Appendix 8). This indicates a positive correlation between increasing deviation of relative mortality and the degree of pattern formation.

This type of correlation is not obligatory, for many curves show that the regional influence modifies the rate to some intermediate value, in which circumstances there will be a negative correlation between pattern formation and increasing deviation.

### VI. TOPOGRAPHICAL DISTRIBUTION OF THE SIGNIFICANTLY PATTERNED AREAS.

Fig. 3 and 5 show the topographical distribution of the counties in the " significant " grades (or intergrades) of Table I and Fig. 2 and 4, including, in addition, the topographical distribution of all counties whose relative mortalities deviate in excess of 2 S.E. (see Appendix 5).

The system of shading employed is explained in Section VII.

While these distributions range from (*a*) scattered isolated counties through (*b*) small and (*c*) large areas of significance to cases where (*d*) the whole 62 counties participate in abnormal pattern formation, the main feature is the frequent occurrence of *large zones* of significant patterning.

The position is summarized in Table VI, which shows that all but three of the maps have one or more large zones of significance. The average size of a major zone is 26 counties (i.e. 2/5ths of map) and of a minor zone 13 counties (i.e. 1/5th

TABLE VI.—*Features of Significant Zones.*

| Number of zones. | Number of maps with. | Mean size number of counties. | | Mean size of separate zones. Major. | Minor. | | Broad summary area of significance. |
|---|---|---|---|---|---|---|---|
| 0 | 3 | 0 | (0) | .. | .. | .. | |
| 1 | 13 | 23 | (1) | 23 | .. | .. | 2/5th of map |
| 2 | 14 | 41 | (2) | 28 | 13 | .. | 3/5ths or more of map |
| 3 | 2 | 54 | (3) | 31 | 16 | 7 | 3/5ths or more of map |
| All | 32 | 31 | | 26(±8) | 13(±8) | 7 | 1/2 of map |

(*N.B.*—Additional data from 2 S.E. deviations in relative mortality *not* here included.)

of map). Generally speaking *2/5ths or more* of the area of the map participate in *significant* pattern formation.

The consistent presence of these larger zones seems to indicate that the *distribution* of Regional Influences evolves around some fairly simple theme. If

FIG. 2.

The "intensity" of a regional influence can be expressed in terms of the "degree of development" of patterns found within its zone of action; the more non-random the pattern the more powerful the influences at work.

The analysis is best shown graphically; the expected random frequency of the 7 grades and 6 inter-grades patterns of each map are plotted out on an inverted logarithmic scale so that "peaks" show high degrees of pattern development—that is, powerful regional influences.

The striking feature of the curves so formed is their essential simplicity. In fact they are almost as simple as the bold geographical zones from which they arise (Fig. 3 and 5). E.g. take No. 1; here we have a peak ($P = \cdot 01$) between grade 5 and 6 falling off on each side to grades 4 and 7. This shows the intensity structure of the black zone on map 1. There is also another peak ($P = \cdot 05$) between grade 1 and 2 falling off towards grade 4. This shows the intensity structure of the single hatched zone on map 1. Clearly, we have two unrelated zones of regional influences, one of which increased and the other of which decreased relative mortality. Moreover, *each zone represents a single influence.*

Again, Nos. 15 and 16 each show two separate peaks of positive regional influences corresponding to two separate black zones on each of maps 15 and 16, and so on. In general we may say that every peak represents a single distinct regional influence.

Peaks marked W show patterns in Welsh counties.

we combine this observation with that of the previous section, viz. that the intensity also follows definite *trends*, we begin to get a clearer picture of the essential attributes of these influences (Appendix 6).

FIG. 3.

Every shaded county on these maps either participates in " significant " pattern formation or has a relative mortality showing " significant " deviation from the mean. They indicate the loci of regional influences. Generally, the shaded counties coalesce into " zones," which means that regional influences have bold and simple geographical distribution, and cover areas large in relation to the size of the single county units. This is a fundamental observation. Zones and counties are shaded in three ways ; see Table below :

| Shading. | Includes grades. | Effect of regional influences. |
|---|---|---|
| Black . . . | 5 6 7 | INCREASES Relative Mortality |
| Double hatch . | 4 | HOLDS at INTERMEDIATE level |
| Single hatch . | 1 2 3 | DECREASES Relative Mortality |

Note particularly the important distinction between mere absence of a significant regional influence (unshaded) and the presence of regional influences actively decreasing mortality (single hatch).

The extent to which each zone represents a " single " regional influence is a matter for more detailed investigation ; one method of approaching this problem is given in the adjoining footnote to Fig. 2.

### VII. CORRELATION OF P TRENDS AND TOPOGRAPHICAL DISTRIBUTIONS.

In comparing corresponding curves and maps in Fig. 2 to 5 note :

1. That the shading of the zone varies with the position of the peak according to the following convention:

Peak at Grade 1, 2, or 3 shading is single hatching.
,, ,, 4 ,, ,, cross hatching.
,, ,, 5, 6, or 7 ,, ,, black.
Unshaded zones have $P > \cdot 05$.

Fig. 4.
Legend as in Fig. 2.

2. That a significant " peak " on the curve ($P < \cdot 05$) is *always* associated with a significant zone on the map.

3. That a significant " zone " is *not always* associated with a significant peak. In such cases the individual counties of the zone each show a deviation from the mean $\geqslant \pm$ 2 S.E. (Stocks' data), but collectively fail to participate in significant pattern formation. 1.5.7.10.19.20.25.28 ; usually such zones are associated with a " sub-critical " peak.

4. That significant peaks are not isolated, but rise naturally from less critically significant patterns of neighbouring grades. Even the low portions of the curves show strong evidence of non-random forms (hill and valley picture).

Correspondingly, significant zones on the map are not isolated, but merge naturally into zones of lower significance ; unfortunately this feature only shows crudely on the maps, as zones are merely dichotomized into significant and non-significant. Fig. 6 gives a truer picture of the type of merging involved.

Fig. 5.
Legend as in Fig. 3.

5. That there are cases in which smooth trend of a curve is abruptly broken by some intermediate peak or minimum. These are generally associated with some degree of " isolation " or independence of the corresponding geographical zone (e.g. Welsh peak). 1.23.26.

6. That curves of type 2 which develop a peak before the extreme grades and then fall off are usually associated with maps in which the highest (or lowest) grades do not form a single zone, but break up into two or more sub-zones, e.g. grade 7 may appear as three separate zones, unrelated to each other, but all related to grade 6, ∴ pattern intergrade 67 very much more developed than the pattern of Grade 7.

7. That Wales is apparently the seat of very intense regional influences, for in 26 of the 32 curves there is a peak wholly or partially due to patterns in that country (marked W).

8. That where maps and curves relate to mixed data and not to a single form of cancer one may expect more complicated curves, as there is a greater likelihood of having multiple " centres " due to the superposition of several independent and unrelated pattern distribution. 15 (2–3) centres.

9. That often *apparently* simple geographical zoning (8, 22, 29, 31) is associated with quite complex curves. This apparent simplicity is due to the fact that Grade 5, 6 and 7 (or 1, 2 and 3) are not differentiated on our maps, and for more detailed study Dr. Stocks' originals should be consulted.

In comparing zones and peaks remember that a peak results from a " significant pattern," and that significant patterns arise from " geographical continuity of allied countries " ; and that this continuity results from a regional influence. Hence every peak represents a regional influence in some specific geographical zone.

### VIII. THE GEOGRAPHICAL UBIQUITY OF REGIONAL INFLUENCES.

The zones of significance, which vary in position from map to map, indicate the loci of influences affecting various forms of cancer. How does the prevalence of these influences vary in different parts of the country ? In terms of the data available, this is equivalent to determining the *frequency* with which *each county* participates in significant pattern formation.

To ensure rough comparability in the data to be examined, we first select from the 32 maps analysed only those dealing with cancer of specific sites (e.g. we discard general categories such as " all forms "—maps 16, 17, 24, 32, 33).

The remaining 28 maps are then comparable in the sense that in each case we are using the relative cancer mortality for some particular site incidence as a test or index of the presence (or absence) of regional influences affecting that particular site.

Table VII shows, on this basis, the frequency (as a fraction of 28) with which each county participates in significant pattern formation, including single counties where the deviation exceeds $\pm$ 2 S.E. The frequency of effects " increasing " and " decreasing " the rate are shown separately ; the frequency of effects " modifying " the rate (significantly) is the sum of these two.

In the absence of regional influences we anticipate that a county will participate in " random " significant pattern formations in about 1/20 cases, i.e. 1·5 times per 28 county appearances. The *lowest* corresponding frequency recorded in Table VII is eight times greater than this (viz. Bedfordshire and Peterborough 12/28). The mean frequencies are, of course, still higher.

We therefore conclude (1) that these regional influences are geographically ubiquitous, and (2) that their prevalence (as measured by the number of forms of cancer they affect significantly) varies in different parts of the country.

### IX. THE COMPOSITE REGIONAL INFLUENCE.

We have seen that while the topographical and intensity trends for individual site incidences are, in general, well defined (i.e. form a hill and valley picture), the zones of one map overlap to a varying degree the zones of another (Section VIII).

TABLE VII.—*The Number of Times (x) Each County Participates in Significant Pattern Formation. (Frequency = x/28.)*

| County. | Increases (I). | Decreases (D). | Modifies (I + D). | County. | Increases (I). | Decreases (D). | Modifies (I + D). |
|---|---|---|---|---|---|---|---|
| | Effect of regional influence on relative mortality. | | | | Effect of regional influence on relative mortality. | | |
| Bedford | 10 | 2 | 12 | Staffordshire | 9 | 8 | 17 |
| Berkshire | 10 | 9 | 19 | Suffolk, East | 10 | 7 | 17 |
| Buckingham | 11 | 8 | 19 | Suffolk, West | 9 | 7 | 16 |
| Cambridge | 10 | 7 | 17 | Surrey | 11 | 10 | 21 |
| Cheshire | 11 | 8 | 19 | Sussex, East | 11 | 10 | 21 |
| Cornwall | 2 | 11 | 13 | Sussex, West | 12 | 7 | 19 |
| Cumberland | 8 | 8 | 16 | Warwick | 13 | 10 | 23 |
| Derby | 4 | 12 | 16 | Westmorland | 13 | 8 | 21 |
| Devon | 7 | 13 | 20 | Wight, Isle of | 9 | 5 | 14 |
| Dorset | 3 | 9 | 12 | Wiltshire | 8 | 8 | 16 |
| Durham | 11 | 15 | 26 | Worcester | 9 | 9 | 18 |
| Ely, Isle of | 13 | 4 | 17 | Yorks, E. Riding | 11 | 10 | 21 |
| Essex | 12 | 8 | 20 | Yorks, N. Riding | 3 | 14 | 17 |
| Gloucester | 7 | 11 | 18 | Yorks, W. Riding | 13 | 9 | 22 |
| Hereford | 5 | 11 | 16 | | | | |
| | | | | WALES. | | | |
| Hertford | 9 | 9 | 18 | Anglesey | 6 | 8 | 14 |
| Huntingdon | 10 | 3 | 13 | Brecknock | 6 | 10 | 16 |
| Kent | 13 | 5 | 18 | Caernarvon | 6 | 10 | 16 |
| Lancashire | 12 | 10 | 22 | Cardigan | 8 | 9 | 17 |
| Leicester | 10 | 7 | 17 | Carmarthen | 6 | 13 | 19 |
| Lincoln, Holland | 13 | 5 | 18 | Denbigh | 9 | 8 | 17 |
| Lincoln, Kesteven | 9 | 9 | 18 | Flint | 15 | 4 | 19 |
| Lincoln, Lindsey | 10 | 12 | 22 | Glamorgan | 5 | 16 | 21 |
| London | 12 | 10 | 22 | Merioneth | 5 | 11 | 16 |
| Middlesex | 15 | 7 | 22 | Monmouth | 4 | 16 | 20 |
| Norfolk | 8 | 6 | 14 | Montgomery | 6 | 8 | 14 |
| Northampton | 10 | 6 | 16 | Pembroke | 7 | 7 | 14 |
| Northumberland | 10 | 9 | 19 | Radnor | 3 | 14 | 17 |
| Nottingham | 10 | 10 | 20 | | | | |
| Oxford | 13 | 5 | 18 | | | | |
| Peterborough | 9 | 3 | 12 | MEAN | 8·94 | 8·71 | 17·65 |
| Rutland | 8 | 6 | 14 | S.D. | 3·14 | 3·0 | 3·0 |
| Shropshire | 6 | 8 | 14 | | | | |
| Somerset | 4 | 10 | 14 | | | | |
| Southampton | 12 | 8 | 20 | | | | |

This table includes single counties whose relative mortality deviates from the mean (= 100 per cent) by $\pm \gg 2 \times$ S.E. (Stocks' data).

Now, if the distributions and trends in each of the 28 maps are unrelated, the result of combining these inter-random distributions should be to flatten out the whole pattern (irregular multiple hillock picture). On the other hand, if they are related, their combination may be expected to result in some definite composite pattern or configuration (hill and valley picture).

From the entries of Table VII three contour maps were prepared (Appendix 7); these are shown in Fig. 6, and it is evident that whether we take the combinations of the distribution of regional influences, increasing, decreasing, or modifying the rates, we still retain well developed " hill and valley " effects.

This suggests that the various individual site patterns show some inter-relationship, or at least possess some " common factor."

(*N.B.*—The composite regional influence here discussed differs from the regional influence as measured in terms of relative mortality from " all causes." The latter deals only with the algebraic sum of plus and minus effects, while the present shows the magnitude of the sum of all effects, irrespective of sign.)

FIG. 6.

This shows the frequency with which regional influences are found in different parts of the country. It summarizes the data of Fig. 3 and 5, e.g. No. 3 sums all the " single hatched " counties and zones on maps 1–33, and indicates that significant influences (or zones) decreasing cancer are most frequent in the south-west and north-east.

Observe the ubiquity of regional influences, and also the general simplicity of their collective distribution.

*Use of contours.*—The area over which a regional influence acts is generally much more extensive than that of a single county (Fig. 3 and 5). Moreover, the intensity of pattern formation throughout this area follows a simple smooth trend (Fig. 2 and 4). We may therefore conclude that county boundaries have no essential significance in relation to regional influences. In fact these influences behave in every way as continuous variables, and their variations in intensity are most appropriately depicted by means of contour-lines on the surface of the map.

To depict these regional influences by contours may serve to drive home another of their characteristics as revealed by this study, viz. that they behave in every respect as " continuous variables." Any concept of discontinuity is an artefact arising from the use of " counties " as pattern units ; county boundaries have no essential significance in relation to regional influences.

### X. THE GENERAL CHARACTERISTICS OF REGIONAL INFLUENCES.

These may now, on the basis of the preceding sections, be summarized as follows :

They are present to such an extent that between 1/5th and 1/3rd of all " patterns " are individually significant, and the collective mean probability of all patterns, including the non-significant, differs from random by at least 9 to

TABLE VIII.—*Effective* P *of Size of Group* (Pnn$_1$). *Gives the Probability of Any Selected Single Group Observed on the Map.* (N.B.—*Grade Pattern*

Size of

| Number of counties in Grade = *n*. | 1. | 2. | 3. | 4. | 5. | 6. | 7. | 8. | 9. | 10. | 11. | 12. | 13. | 14. | 15. | 16. | 17. | 18. |
|---|---|---|---|---|---|---|---|---|---|---|---|---|---|---|---|---|---|---|
| 2 | 100 | 8 | | | | | | | | | | | | | | | | |
| 3 | 100 | 19 | 1 | | | | | | | | | | | | | | | |
| 4 | 100 | 38 | 5 | * | | | | | | | | | | | | | | |
| 5 | 100 | 63 | 7 | 1 | * | | | | | | | | | | | | | |
| 6 | 100 | 89 | 18 | 6 | * | * | | | | | | | | | | | | |
| 7 | 100 | 100 | 31 | 10 | 1 | * | * | | | | | | | | | | | |
| 8 | 100 | 100 | 47 | 15 | 4 | * | * | * | | | | | | | | | | |
| 9 | 100 | 100 | 59 | 22 | 7 | 2 | * | * | * | | | | | | | | | |
| 10 | 100 | 100 | 72 | 31 | 16 | 6 | 1 | * | * | * | | | | | | | | |
| 11 | 100 | 100 | 89 | 43 | 24 | 9 | 2 | 1 | 1 | * | * | | | | | | | |
| 12 | 100 | 100 | 100 | 52 | 30 | 12 | 4 | 1 | 1 | 1 | * | * | | | | | | |
| 13 | 100 | 100 | 100 | 64 | 37 | 16 | 7 | 4 | 2 | 1 | * | * | * | | | | | |
| 14 | 100 | 100 | 100 | 77 | 48 | 21 | 9 | 6 | 4 | 3 | 2 | 1 | * | * | | | | |
| 15 | 100 | 100 | 100 | 93 | 56 | 28 | 17 | 10 | 6 | 4 | 3 | 2 | 1 | * | * | | | |
| 16 | 100 | 100 | 100 | 100 | 69 | 38 | 25 | 18 | 11 | 5 | 4 | 3 | 2 | 1 | * | * | | |
| 17 | 100 | 100 | 100 | 100 | 82 | 43 | 30 | 24 | 18 | 11 | 5 | 4 | 2 | 1 | * | * | * | |
| 18 | 100 | 100 | 100 | 100 | 91 | 53 | 38 | 29 | 24 | 17 | 11 | 6 | 5 | 3 | 1 | * | * | * |
| 19 | 100 | 100 | 100 | 100 | 100 | 70 | 48 | 35 | 30 | 23 | 14 | 9 | 6 | 4 | 2 | 1 | * | * |
| 20 | 100 | 100 | 100 | 100 | 100 | 80 | 59 | 41 | 34 | 28 | 19 | 14 | 10 | 7 | 4 | 2 | * | * |
| 21 | 100 | 100 | 100 | 100 | 100 | 93 | 66 | 47 | 41 | 34 | 26 | 21 | 17 | 13 | 10 | 6 | 3 | 2 |
| 22 | 100 | 100 | 100 | 100 | 100 | 100 | 81 | 65 | 54 | 43 | 33 | 26 | 22 | 18 | 13 | 8 | 4 | 3 |
| 23 | X | X | X | X | X | X | X | X | X | X | 45 | 37 | 28 | 23 | 19 | 15 | 10 | 6 |
| 24 | X | X | X | X | X | X | X | X | X | X | X | 40 | 30 | 24 | 19 | 16 | 12 | 8 |
| 25 | X | X | X | X | X | X | X | X | X | X | X | X | 40 | 33 | 26 | 20 | 15 | 11 |
| 26 | X | X | X | X | X | X | X | X | X | X | X | X | X | 40 | 31 | 24 | 20 | 15 |
| 27 | X | X | X | X | X | X | X | X | X | X | X | X | X | X | X | X | 30 | 25 |
| 28 | X | X | X | X | X | X | X | X | X | X | X | X | X | X | X | X | X | X |
| 29 | X | X | X | X | X | X | X | X | X | X | X | X | X | X | X | X | X | X |
| 30 | X | X | X | X | X | X | X | X | X | X | X | X | X | X | X | X | 33 | 26 |
| 31 | X | X | X | X | X | X | X | X | X | X | X | X | X | X | 39 | 39 | 39 | 39 |
| 32 | X | X | X | X | X | X | X | X | X | X | X | 63 | 63 | 63 | 63 | 63 | 63 | 63 |
| 33 | X | X | X | X | X | X | X | X | X | 66 | 66 | 66 | 66 | 66 | 66 | 66 | 66 | 66 |
| 34 | 100 | 100 | 100 | 100 | 100 | 89 | 81 | 73 | 67 | 67 | 67 | 67 | 67 | 67 | 67 | 67 | 67 | 67 |
| 35 | 100 | 100 | 100 | 100 | 90 | 79 | 70 | 64 | 64 | 64 | 64 | 64 | 64 | 64 | 64 | 64 | 64 | 64 |
| 36 | 100 | 100 | 100 | 89 | 81 | 69 | 60 | 60 | 60 | 60 | 60 | 60 | 60 | 60 | 60 | 60 | 60 | 60 |
| 37 | 100 | 100 | 100 | 80 | 72 | 64 | 57 | 57 | 57 | 57 | 57 | 57 | 57 | 57 | 57 | 57 | 57 | 57 |
| 38 | 100 | 100 | 100 | 78 | 71 | 61 | 54 | 54 | 54 | 54 | 54 | 54 | 54 | 54 | 54 | 54 | 54 | 54 |
| 39 | 100 | 100 | 96 | 74 | 66 | 58 | 51 | 51 | 51 | 51 | 51 | 51 | 51 | 51 | 51 | 51 | 51 | 51 |
| 40 | 100 | 100 | 73 | 53 | 46 | 39 | 33 | 33 | 33 | 33 | 33 | 33 | 33 | 33 | 33 | 33 | 33 | 33 |

To find the effective proba

1. Divide tabulated

2. Call " X " $P = <\cdot 01$.

3. Call " * " $P = >\cdot 5$.

18 times the S.E. Significant patterns can be demonstrated in all parts of England and Wales, their *minimum* presence (or frequency) being still eight times greater than random.

The intensity of their effect usually varies in a simple and regular fashion both between consecutive grades and across contiguous geographical areas ; i.e. they behave as " continuous variables " and their effects are most appropriately represented on maps by means of contour lines.

As operative on any particular form of cancer regional influences appear to be few rather than many in number and developed over wide rather than over small tracts.

Finally, although the regional influences affecting different forms of cancer each have their own distribution, all are apparently related to some common key

*)btaining, by Chance, a Single Group of Counties Equal to or Larger in* Size *than*
*:an be Assessed in Terms of the* $Pnn_1$ *of the Largest Single Group Present.)*

| | 19. | 20. | 21. | 22. | 23. | 24. | 25. | 26. | 27. | 28. | 29. | 30. | 31. | 32. | 23. | 34. | 35. | 36. | 37. | 38. | 39. | 40. |
|---|---|---|---|---|---|---|---|---|---|---|---|---|---|---|---|---|---|---|---|---|---|---|
| }roup = $n_1$. | | | | | | | . | | | | | . | | | | | . | | | | | . |
| | | . | | | | | . | | | | | . | | | | | . | | | | | . |
| | | . | | | | | . | | | | | . | | | | | . | | | | | . |
| | | . | | | | | . | | | | | . | | | | | . | | | | | . |
| | | . | | | | | . | | | | | . | | | | | . | | | | | . |
| | | . | | | | | . | | | | | . | | | | | . | | | | | . |
| | | . | | | | | . | | | | | . | | | | | . | | | | | . |
| | | . | | | | | . | | | | | . | | | | | . | | | | | . |
| | | . | | | | | . | | | | | . | | | | | . | | | | | . |
| | | . | | | | | . | | | | | . | | | | | . | | | | | . |
| | | . | | | | | . | | | | | . | | | | | . | | | | | . |
| | | . | | | | | . | | | | | . | | | | | . | | | | | . |
| | | . | | | | | . | | | | | . | | | | | . | | | | | . |
| | | . | | | | | . | | | | | . | | | | | . | | | | | . |
| | | . | | | | | . | | | | | . | | | | | . | | | | | . |
| | | . | | | | | . | | | | | . | | | | | . | | | | | . |
| | | . | | | | | . | | | | | . | | | | | . | | | | | . |
| | * | . | | | | | . | | | | | . | | | | | . | | | | | . |
| | * | * | | | | | . | | | | | . | | | | | . | | | | | . |
| | 1 | * | * | | | | . | | | | | . | | | | | . | | | | | . |
| | 2 | 1 | * | * | | | . | | | | | . | | | | | . | | | | | . |
| | 4 | 2 | 1 | * | * | | . | | | | | . | | | | | . | | | | | . |
| | 5 | 3 | 2 | 1 | * | * | . | | | | | . | | | | | . | | | | | . |
| | 8 | 6 | 4 | 2 | 1 | * | * | | | | | . | | | | | . | | | | | . |
| | 12 | 9 | 7 | 5 | 3 | 1 | * | * | | | | . | | | | | . | | | | | . |
| | 19 | 14 | 10 | 7 | 4 | 2 | 1 | * | * | | | . | | | | | . | | | | | . |
| | X | 24 | 19 | 12 | 8 | 5 | 3 | 1 | * | * | | . | | | | | . | | | | | . |
| | X | 26 | 22 | 20 | 15 | 10 | 6 | 3 | 1 | * | * | . | | | | | . | | | | | . |
| | 26 | 26 | 26 | 26 | 24 | 19 | 14 | 9 | 5 | 3 | 1 | 1 | | | | | . | | | | | . |
| | 39 | 39 | 39 | 39 | 39 | 36 | 34 | 25 | 17 | 11 | 5 | 2 | 1 | | | | . | | | | | . |
| | 63 | 63 | 63 | 63 | 63 | 63 | 49 | 35 | 27 | 19 | 11 | 7 | 4 | 2 | | | . | | | | | . |
| | 66 | 66 | 66 | 66 | 66 | 66 | 66 | 55 | 45 | 35 | 27 | 19 | 11 | 6 | 3 | | . | | | | | . |
| | 67 | 67 | 67 | 67 | 67 | 67 | 67 | 67 | 58 | 49 | 40 | 31 | 20 | 13 | 7 | 3 | . | | | | | . |
| | 64 | 64 | 64 | 64 | 64 | 64 | 64 | 64 | 64 | 58 | 51 | 44 | 37 | 24 | 16 | 9 | 4 | | | | | . |
| | 60 | 60 | 60 | 60 | 60 | 60 | 60 | 60 | 60 | 60 | 57 | 52 | 47 | 42 | 30 | 20 | 11 | 4 | | | | . |
| | 57 | 57 | 57 | 57 | 57 | 57 | 57 | 57 | 57 | 57 | 57 | 55 | 51 | 47 | 43 | 30 | 19 | 11 | 4 | | | . |
| | 54 | 54 | 54 | 54 | 54 | 54 | 54 | 54 | 54 | 54 | 54 | 54 | 54 | 51 | 48 | 45 | 34 | 25 | 15 | 5 | | . |
| | 51 | 51 | 51 | 51 | 51 | 51 | 51 | 51 | 51 | 51 | 51 | 51 | 51 | 51 | 51 | 50 | 49 | 40 | 29 | 19 | 6 | . |
| | 33 | 33 | 33 | 33 | 33 | 33 | 33 | 33 | 33 | 33 | 33 | 33 | 33 | 33 | 33 | 41 | 49 | 58 | 46 | 33 | 20 | 7 |

bility of size of Group $Pnn_1$ :

value by 100.

pattern. They are not the cause of cancer, but impress their effect upon a general non-regional factor which operates to produce a definite positive incidence of cancer in all parts of England and Wales.

### APPENDIX 1.

### CALCULATING PATTERN PROBABILITY.

It is shown by Cruickshank (1940) that in a grade of $n$ counties where $n_1$ = the number of counties in the " group " to be analysed, $g$ = the total number of groups into which the $n$ counties arrange themselves, then reference to Table VIII under arguments $n$ and $n_1$ will give the probability $Pnn_1$ of obtaining, by pure chance, a group of $n_1$ *or larger* ; and reference to Table IX under arguments $n$ and $g$ will give the probability $Png$ that the $n$ counties are arranging themselves, by pure chance, *into g or some smaller* number of groups.

TABLE IX.—*Effective* P *of Number of Groups* (Png). *Gives the Probability that the Counties in Any Selected Grade will Arrange themselves by Chance into an Equal or Smaller* Number *of Groups than Observed on the Map.*

| Number of counties = "n." | 1. | 2. | 3. | 4. | 5. | 6. | 7. | 8. | 9. | 10. | 11. | 12. | 13. | 14. | 15. |
|---|---|---|---|---|---|---|---|---|---|---|---|---|---|---|---|
| | | | | | Number of Groups in the Grade = " g." | | | | | | | | | | |
| 2 | 8 | 100 | | | | | | | | | | | | | |
| 3 | 1 | 19 | 100 | | | | | | | | | | | | |
| 4 | * | 5 | 38 | 100 | | | | | | | | | | | |
| 5 | * | 2 | 11 | 58 | 100 | | | | | | | | | | |
| 6 | * | 1 | 6 | 34 | 71 | 100 | | | | | | | | | |
| 7 | * | 1 | 2 | 18 | 52 | 82 | 100 | | | | | | | | |
| 8 | * | * | 3 | 13 | 34 | 66 | 91 | 100 | | | | | | | |
| 9 | * | * | 3 | 7 | 29 | 52 | 78 | 94 | 100 | | | | | | |
| 10 | * | * | 2 | 7 | 22 | 44 | 67 | 89 | 98 | 100 | | | | | |
| 11 | * | * | 2 | 4 | 14 | 35 | 62 | 83 | 96 | 100 | 100 | | | | |
| 12 | * | * | 2 | 3 | 15 | 27 | 53 | 77 | 92 | 98 | 100 | 100 | | | |
| 13 | * | * | 2 | 5 | 13 | 25 | 46 | 74 | 83 | 94 | 98 | 100 | 100 | | |
| 14 | * | * | 1 | 3 | 10 | 25 | 51 | 67 | 80 | 89 | 97 | 99 | 100 | 100 | |
| 15 | * | * | 2 | 4 | 6 | 22 | 47 | 62 | 79 | 88 | 96 | 99 | 100 | 100 | 100 |
| 16 | * | * | 2 | 4 | 9 | 22 | 47 | 63 | 78 | 90 | 95 | 99 | 100 | 100 | 100 |
| 17 | * | * | 2 | 5 | 11 | 21 | 45 | 68 | 77 | 87 | 93 | 99 | 100 | 100 | 100 |
| 18 | * | * | 2 | 4 | 13 | 23 | 40 | 63 | 76 | 87 | 95 | 98 | 100 | 100 | 100 |
| 19 | * | * | 1 | 3 | 12 | 22 | 44 | 61 | 79 | 87 | 94 | 100 | 100 | 100 | 100 |
| 20 | * | * | 1 | 3 | 11 | 25 | 46 | 60 | 80 | 88 | 94 | 99 | 100 | 100 | 100 |
| 21 | * | * | 1 | 3 | 10 | 25 | 48 | 69 | 83 | 94 | 97 | 98 | 99 | 100 | 100 |
| 22 | * | * | 1 | 4 | 13 | 27 | 46 | 71 | 82 | 94 | 97 | 99 | 99 | 100 | 100 |
| 23 | * | * | 2 | 7 | 16 | 29 | 55 | 70 | 88 | 96 | 97 | 99 | 99 | 99 | 100 |
| 24 | * | * | 1 | 5 | 13 | 34 | 57 | 74 | 89 | 97 | 98 | 98 | 100 | 100 | 100 |
| 25 | * | * | 3 | 8 | 19 | 40 | 60 | 74 | 92 | 97 | 98 | 98 | 100 | 100 | 100 |
| 26 | * | * | 3 | 7 | 24 | 45 | 60 | 81 | 92 | 96 | 97 | 99 | 99 | 100 | 100 |
| 27 | * | 1 | 4 | 14 | 29 | 51 | 67 | 82 | 94 | 96 | 96 | 99 | 100 | 100 | 100 |
| 28 | * | 2 | 6 | 19 | 33 | 55 | 72 | 87 | 95 | 96 | 98 | 100 | 100 | 100 | 100 |
| 29 | 1 | 2 | 8 | 21 | 35 | 60 | 80 | 87 | 96 | 97 | 98 | 100 | 100 | 100 | 100 |
| 30 | 1 | 2 | 9 | 22 | 38 | 69 | 84 | 90 | 96 | 97 | 98 | 100 | 100 | 100 | 100 |
| 31 | 2 | 5 | 12 | 26 | 44 | 75 | 88 | 91 | 97 | 98 | 99 | 99 | 100 | 100 | 100 |
| 32 | 2 | 5 | 15 | 37 | 59 | 80 | 91 | 94 | 96 | 97 | 99 | 99 | 100 | 100 | |
| 33 | 3 | 7 | 26 | 45 | 67 | 83 | 91 | 95 | 97 | 98 | 100 | 100 | 100 | | |
| 34 | 4 | 9 | 27 | 49 | 73 | 89 | 93 | 96 | 98 | 100 | 100 | 100 | | | |
| 35 | 4 | 11 | 29 | 55 | 77 | 86 | 93 | 95 | 99 | 100 | 100 | | | | |
| 36 | 4 | 17 | 38 | 60 | 82 | 91 | 96 | 98 | 100 | 100 | | | | | |
| 37 | 5 | 17 | 41 | 64 | 85 | 96 | 98 | 100 | 100 | | | | | | |
| 38 | 4 | 22 | 46 | 69 | 89 | 95 | 99 | 100 | | | | | | | |
| 39 | 6 | 26 | 52 | 77 | 90 | 99 | 100 | | | | | | | | |
| 40 | 7 | 29 | 58 | 83 | 95 | 100 | | | | | | | | | |

To find the effective probability of the number of groups *Png*—

1. Divide the tabulated value by 100.
2. Call * $P = < \cdot 01$.

The analysis is entirely automatic (see Appendix 2), and no judgment is required other than deciding whether or not counties are actually " in contact," so forming " a group." This is usually perfectly obvious, but there are a few doubtful instances which are dealt with by the arbitrary standardization shown in Fig. 7.

FIG. 7.

This map shows how the " contact number " varies from county to county, e.g. for Devon this is 3 as the county " contacts " three others, viz. Cornwall, Somerset and Dorset. While the majority of counties meet at a common county boundary, occasionally they meet only over a very narrow range, the so-called " point contacts " (solid circles on map). Where the county boundary is a river or an estuary the counties may fail to make a *de facto* contact, and yet, it may be necessary to consider these as " touching " for the purpose of studying regional influences (solid rectangles on map).

Note the distinctly higher values of the contact numbers of the inland counties as contrasted with those of the coastal counties.

The mean contact number (C) works out at 5·12. This is an important constant in theoretical formulae, e.g. the expectation of obtaining " pairs " with $\frac{n}{N}$ counties occupied is $\frac{C(n \times n - 1)}{2(N - 1)}$ and so on.

### APPENDIX 2.

### MECHANICAL ANALYSIS OF PATTERNS.

A " mirror-outline " county map is drawn with a "'glass-ink " on the undersurface of a sheet of glass. Invert and place upon and congruent with map to be analysed. Mark on the glass with grease pencil all " Grade 1 " counties. Remove glass template and place on white sheet. Analyse arrangement of marked counties ; record ; clean off wax pencil mark with chloroform. Replace on map and proceed similarly to work and analyse " Grade 2 " counties, and so on. By this means grades are presented for examination free of confusing context. It is helpful to indicate permanently on the template the arbitrary decisions *re* contacts, i.e. to make it look in all regards as Fig. 7.

Intergrading is carried out by appropriate modification of the above procedure. Records are entered on the special analysis sheets (Appendix 3).

### APPENDIX 3.

### RECORDING PATTERNS.

### *Sample Analysis Sheet* (Map No. 17).

| Grade. Number | Grade. Number in "n" | Size of groups "n"$_1$. 1. | 2. | 3. | 4. | 5. | 6. | 7. | 8. | 9. | 10. | 11. | 12. | 13. | 14. | > 14. | Number of Groups "g." |
|---|---|---|---|---|---|---|---|---|---|---|---|---|---|---|---|---|---|
| 1 | 2 | // | .. | .. | .. | .. | .. | .. | .. | .. | .. | .. | .. | .. | .. | ... | 2 |
| 2 | 10 | //// | / | .. | / | .. | .. | .. | .. | .. | .. | .. | .. | .. | .. | ... | 6 |
| 3 | 20 | /// | .. | / | .. | / | .. | .. | .. | / | .. | .. | .. | .. | .. | ... | 6 |
| 4 | 19 | // | // | // | .. | .. | .. | / | .. | .. | .. | .. | .. | .. | .. | ... | 7 |
| 5 | 17 | // | .. | .. | .. | / | .. | .. | .. | .. | .. | .. | .. | .. | .. | ... | 3 |
| 6 | 2 | .. | / | .. | .. | .. | .. | .. | .. | .. | .. | .. | .. | .. | .. | ... | 1 |
| 7 | 2 | // | .. | .. | .. | .. | .. | .. | .. | .. | .. | .. | .. | .. | .. | ... | 2 |
| | 62 | 15 | 4 | 3 | 1 | 2 | * | 1 | * | 1 | * | * | * | * | * | * | 27 |

Check $\Sigma\, n = 62$ (always). $\Sigma$ columns + $\Sigma$ rows + 27 (in this case) :

$(15 \times 1) + (4 \times 2) + (3 \times 3)$ +) $(1 \times 4) + (2 \times 5) + (1 \times 7) + (1 \times 9) = 62$ (always.)

The $P$ values corresponding to the various $ng$ (and/or $nn_1$) entries are read off from Tables IX (and/or VIII) and finally collected in a summary sheet.

### *Sample Summary Sheet* (Map No. 17).

| Grade. | 1. | 2. | 3. | 4. | 5. | 6. | 7. | |
|---|---|---|---|---|---|---|---|---|
| *ng* | 2;2 | 10;6 | 20;6 | 19;7 | 7;3 | 2;1 | 2;2 | (*) |
| *Png* | *1·00* | *·44* | *·25* | *·44* | *·02* | *·08* | *1·00* | |
| $nn_1$ | 2;1 | 10;4 | 20;9 | 19;7 | 7;5 | 2;2 | 2;1 | |
| $Pnn_1$ (if < $Png$) | .. | .. | (·31) | .. | (01) | .. | .. | (†) |

(*) i.e. Second and final column entries of analysis sheet.

(†) i.e. Second column and highest recorded value of corresponding $n_1$.

Intergrade analyses are entered on similar sheets.

### APPENDIX 4.

### HIGHER PATTERNING OF INTERGRADES.

The 192 intergrades are composed entirely of the 210 grades. Each intergrade, being composed of two grades, is, on the average, just twice the size of the individual constituent grades. The patterns in these two units are analysed by a method which fully compensates for difference in size. Hence, we might anticipate that the means for these two " sampling " series (i.e. of the 192 intergrade- and 210 grade-patterns) *should* show good agreement. That this is not the case calls for some explanation.

The map pattern contains 62 elements (counties). To formalize take the analogous special case of an 8 × 8 " map " with the basic pattern shown in Fig. 8. Then the random probability of any *fragment* (or grade) of this pattern is greater than that of a larger fragment (or intergrade) and still greater than that of the whole, e.g.—

1/4 fragment (16 squares) $P_{16} = \dfrac{13!\ 3!}{16!} = \cdot 1785 \times 10^{-2}$

1/2 fragment (32 squares) $P_{32} = \dfrac{26!\ 6!}{32!} = \cdot 1104 \times 10^{-5}$

whole (64 squares) $P_{64} = \dfrac{52!\ 12!}{64!} = \cdot 1269 \times 10^{-12}$

and $P_6 > P_{32} > P_{64}$.

FIG. 8.

It is an interesting fact that the *larger* the area we select for examination the *higher* is the degree of patterning found. As this is not due to the method, which fully compensates for variations in the size of the area examined, it must be attributed to some intrinsic feature of the Regional Influences.

Fig. 8, in conjunction with Appendix 4, indicates the probable, simple explanation, viz. that estimates of the degree of pattern development of an *organized* pattern must generally fall below the true value when they are based on the examination of only part of the pattern—for the simple reason that the relation of one " piece " of the pattern to a neighbouring " piece " is thereby overlooked. The process of " intergrading " was evolved to deal with this contingency.

Thus, to explain the observed fact that the *P* grade greater than *P* intergrade we must assume that the mean " pattern " associated with regional influences covers an area greater than that of a mean grade, i.e. greater than 9 counties $(\frac{62}{7})$. In terms of area this means $>940$ sq. miles.

### APPENDIX 5.

### SIGNIFICANT DEVIATIONS OF RELATIVE MORTALITY.

Where, for example, there is only one county in a grade (or intergrade) pattern formation becomes impossible and *Png* and $Pnn_1$ must both $= 1{\cdot}00$ ; such cases are *italicized* in Table I. But the relative mortality for such a " single " county may deviate so markedly from the mean (i.e. exceed 2 S.E.) that regional influences may be assumed operative. Hence, in cases where a qualitative result only is required (i.e. significant regional influence " present " or " absent ") it is natural to use this supplementary—and orthodox—source of information. It is readily obtainable, as Dr. Stocks classifies all his data with reference to deviations from S.E., viz. as being 0–1, 1–2, and 2 + times S.E.

### APPENDIX 6.

### VISUALIZING COMBINED DATA OF SECTIONS V AND VI.

These zones of significant patterning are not " isolated," but merge with zones of high, though not significant, pattern. These in turn merge with zones of lower pattern. That is, they show geographical trends.

The easiest way to integrate the *P* trends of Section III and the geographical trends of Section IV is to start with a two-dimensional graded map and imagine the level of each county raised in proportion to the reciprocal of the *P* values for the grade (and/or intergrade) to which it belongs. This will give a three-dimensional topographical map, whose surface undulations reflect variations in pattern intensity (i.e. by previous definition, variations in the intensity of regional influences), and whose surface shadings show the distribution of the grades.

When we have made this integration we find that the surface of the map is developed into a few well-marked hills and valleys, the " peaks " of these hills representing areas of " significant " patterning.

### APPENDIX 7.

### PREPARING CONTOUR MAPS.

A county map was pasted on a board. Metal pins, corresponding in height to the frequencies in Table VII were driven vertically into the centre of each county; the whole was then built up with plasticine to the level of the top of the pins. A scriber-gauge, adjustable to various heights, was attached to a pantograph. With this apparatus the contours of the three-dimensional model were automatically transferred to the two-dimensional map-surface.

### APPENDIX 8.

### NEGATIVE REGIONAL INFLUENCES.

There is definite evidence that " negative " influences, i.e. regional influences decreasing cancer exist alongside the " positive," in fact these two types of influence, both of which are represented by peaks and zones, occur with equal frequency (Table VII). Negative effects must not be regarded as merely due to the absence of positive effects, for the observed type of trend and degree of patterning associated with negative zones is of a much higher order than is to be expected from the mere absence of positive effect.

It seems that there must be some general factor *other than regional* which operates to produce a definite incidence of cancer. Regional influences are not themselves the cause of cancer, but merely impress their effect, positively or negatively, upon this general factor.

I wish to express my thanks to Mr. N. R. Ling for preparing the various diagrams.

### REFERENCE.

CRUICKSHANK, D. B.—(1940) *Papworth Res. Bull.*, p. 36.